# Complexes of Zinc-Coordinated Heteroaromatic N-Oxides with Pyrene: Lewis Acid Effects on the Multicenter Donor–Acceptor Bonding

**DOI:** 10.3390/molecules29143305

**Published:** 2024-07-13

**Authors:** Yakov P. Nizhnik, Erin Hansen, Cayden Howard, Matthias Zeller, Sergiy V. Rosokha

**Affiliations:** 1 BioStone, 2815 Exchange Blvd., Southlake, TX 76092, USA; iakovnizhnik@gmail.com; 2 Department of Chemistry, Ball State University, Muncie, IN 47306, USA; erin.hansen@bsu.edu (E.H.); cayden.howard@bsu.edu (C.H.); 3 Department of Chemistry, Purdue University, West Lafayette, IN 47907, USA; zeller4@purdue.edu

**Keywords:** N-oxides, Lewis acids, donor–acceptor complexes, X-ray crystallography, UV-Vis spectroscopy, DFT calculations

## Abstract

4-Nitroquinoline-N-oxide (NQO) and 4-nitropyridine-N-oxide (NPO) are important precursors for the synthesis of substituted heterocycles while NQO is a popular model mutagen and carcinogen broadly used in cancer research; intermolecular interactions are critical for their reactions or functioning in vivo. Herein, the effects of the coordination of N-oxide’s oxygen atom to Lewis acids on multicenter donor–acceptor bonding were explored via a combination of experimental and computational studies of the complexes of NQO and NPO with a typical π-electron donor, pyrene. Coordination with ZnCl_2_ increased the positive electrostatic potentials on the surfaces of these π-acceptors and lowered the energy of their LUMO. Analogous effects were observed upon the protonation of the N-oxides’ oxygen or bonding with boron trifluoride. The interaction of ZnCl_2_, NPO, or NQO and pyrene resulted in the formation of dark co-crystals comprising π-stacked Zn-coordinated N-oxides and pyrene similar to that found with protonated or (reported earlier) BF_3_-bonded N-oxides. Computational studies indicated that the coordination of N-oxides to zinc(II), BF_3_, or protonation led to the strengthening of the multicenter bonding of the nitro-heterocycle with pyrene, and this effect was related both to the increased electrostatic attraction and molecular–orbital interactions in their complexes.

## 1. Introduction

**Scheme 1:**
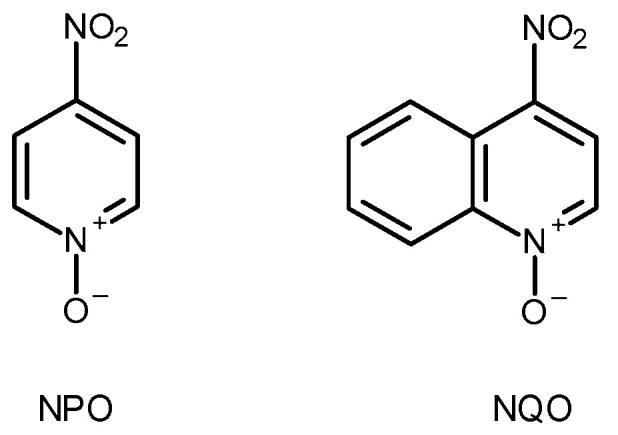
Structures and acronyms of heteroaromatic N-oxides.

Heteroaromatic N-oxides have attracted significant attention from the scientific community due to their extensive synthetic applications and biological activity [1,2,3]. Depending on the nature of the functional group in the heterocycle, the N-oxide group can exhibit electron-donating or electron-withdrawing effects, and therefore heteroaromatic N-oxides are more active in both aromatic electrophilic and nucleophilic substitution reactions than their parent heterocycles. Nitro-substituted N-oxides, 4-nitropyridine-N-oxide, NPO, and 4-nitroquinoline-N-oxide, NQO (Scheme 1) are of special interest in this respect. These substances can be easily obtained from their corresponding pyridine and quinoline N-oxides by nitration, and the nitro group is relatively easily converted into other functional groups leading to a wide variety of substituted heterocycles [2,4].

Besides their synthetic significance, the nitro-derivatives of heteroaromatic N-oxides, especially 4-nitroquinoline-N-oxide, are characterized by strong biological activity. Since the discovery of the mutagenic and carcinogenic activity of NQO in the 1950s (soon after its first preparation), this molecule became a popular model carcinogen and mutagen agent used for the study of carcinogenesis mechanisms, artificial tumorigenesis, and mutant organism production [5,6,7]. 

The presence of the nitro group makes NQO and NPO molecules mild electron π-acceptors (π-acid) suitable for the charge-transfer interactions with planar aromatic electron donors (π-bases) [8,9]. The formation of charge-transfer complexes with DNA and proteins is considered an important factor in the biological action of NQO [10,11,12]. On the other hand, the oxygen atoms in the N-oxide groups are well-suited to interact with Lewis acids [8,13,14,15]. Our earlier spectral and structural studies showed that the coordination of this oxygen to boron trifluoride facilitates the formation of π–π bonded complexes of N-oxides with planar organic donors in the solid state and solutions [8]. Such coordination also substantially enhanced the electron-acceptor properties of the N-oxides, and it facilitated the oxidation of many organic donors (which did not proceed without the Lewis acid) [8]. A computational study by Frontera et al. showed that the halogen, hydrogen, or triel bonding or coordination of the oxygen to a metal ion enhanced the positive potential (π-hole) on the surface of NPO above the C-NO_2_ fragment and, therefore, strengthened the interaction between the N-oxide and various bases [15]. It was also shown that interaction with the Lewis acids substantially accelerates the S_N_Ar reactions of N-oxides [16,17,18,19]. 

The major effects of boron trifluoride on the interaction of N-oxides with organic donors and their redox reactions suggested that Lewis acids could be an important factor controlling the chemical transformations of these molecules and their behavior in living cells. However, while BF_3_ is a common reagent for organic synthesis, its high reactivity precludes its presence or use in biological systems. This raises the question about the effects of other Lewis acids on the intermolecular interactions of aromatic N-oxides, in particular the formation of π-stacked donor–acceptor complexes. Such bonding affects the reactivity of N-oxides [17], and the formation of such complexes is of significant interest for the co-crystallization of pharmaceutical compounds as well as non-linear optical and other materials’ science applications [20,21,22,23]. Thus, in the current work, we explored the effects of the coordination of NQO and NPO to zinc chloride and their protonation on the interaction of these N-oxides with a common organic π-donor, pyrene. Indeed, zinc ions and protons are very common Lewis acids that are found in many chemical and/or biological systems. Furthermore, the crystallographic literature comprises many structures of N-oxide molecules coordinated to zinc(II) halides (or other divalent cations) [24,25,26,27]. Such complexes showed interesting catalytic properties [28]. There are also several structures showing protonated N-oxide molecules (which frequently form hydrogen-bonded dimers with more or less symmetric O-H-O fragments) [29,30,31]. However, there are essentially no data about the effects of such zinc coordination or protonation on the properties of the nitro substituted N-oxides or the interactions of the latter with organic π-donors in solution or the solid states. 

Accordingly, we report herein the results of the structural characterization of co-crystals comprising π–π bonded associations between zinc-coordinated or protonated N-oxides and pyrenes, as well as the results of experimental and computational studies on the effects of the bonding with ZnCl_2_ or protons on the properties of the nitro-substituted N-oxides, and on the multicenter donor–acceptor bonding. Comparison with previously reported data about similar systems with boron trifluoride allows us to elucidate the general effects of Lewis acid on the intermolecular multicenter interactions of the heteroaromatic N-oxides with organic π-donors.

## 2. Results and Discussion

### 2.1. UV-Vis Measurements of Coordination of N-Oxides to Zinc(II) in Solutions 

**Figure 1:**
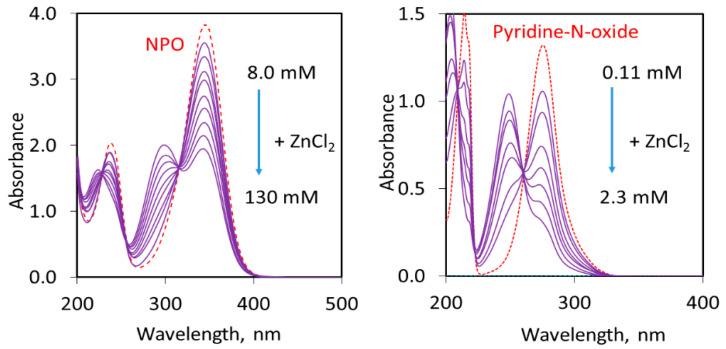
Spectra of acetonitrile solutions with constant concentrations of NPO (2.7 mM, **left**) and pyridine-N-oxide (5.6 mM, **right**) and various concentrations of ZnCl_2_. The spectra of the solutions of individual N-oxides are shown as dashed red lines (the absorption of ZnCl_2_ is negligible in this range).

To evaluate the propensity of zinc(II) to coordinate to heteroaromatic N-oxides, we first carried out the UV-Vis measurements of their interaction in solutions. An addition of ZnCl_2_ to a solution of heteroaromatic N-oxides in acetonitrile resulted in a decrease in the intensity of the absorption bands of the N-oxide molecule and the appearance of new bands at lower wavelengths (Figure 1 and Figure S1 in the Supporting Information).

A similar appearance of blue-shifted absorption bands was observed upon the protonation of the N-oxides (see Figure S2 in the Supporting Information). Also, analogous spectral changes were reported earlier upon the addition of BF_3_ [8]. 

The UV-Vis measurements with different N-oxides showed that the strength of their bonding with zinc(II) is related to the basicity of the N-oxide oxygens which is determined by the electron-withdrawing or donating abilities of the substituents [32]. Indeed, the addition of ZnCl_2_ to the pyridine-N-oxide (Figure 1) or its p-methoxy substituted analogs (Figure S1) in a 1:2 molar ratio resulted in the essentially complete disappearance of the original absorption bands of the N-oxide molecule and the formation of new bands. This suggests strong 2:1 bonding of these N-oxides to zinc(II). In comparison, a substantial amount of the uncoordinated NPO and NQO molecules remain in solutions even after the addition of a large excess of ZnCl_2_ (Figure 1 and Figure S1 in the Supporting Information). This trend is similar to that observed upon the addition of HPF_6_ to the solution of various N-oxides, as well as an earlier reported variation in the formation constants of the halogen-bonded complexes of diiodine with these molecules [13,14,33]. (Note that the determinations of the formation constants of 1:1 and 1:2 complexes of zinc(II) with N-oxides would require additional investigations that are beyond the scope of the current manuscript).

An addition of pyrene to the solutions containing nitro-substituted N-oxides in acetonitrile, dichloromethane, or THF resulted in a change in color from yellow to orange (Figure S3 in the Supporting Information). Earlier studies related these spectral changes to the formation of charge-transfer complexes between the π-acceptors (NPO or NQO) and the π-donor, pyrene [8]. The addition of ZnCl_2_ to these solutions resulted in an immediate change in the color to brown-red. (Figure S3 in the Supporting Information). Similar color changes were observed earlier upon the addition of pyrene to the solutions of BF_3_-coordinated NPO and NQO molecules, and they were related to the formation of charge-transfer complexes between BF_3_-coordinated NPO or NQO and pyrene. These complexes showed absorption bands in the 500–600 nm range (which are red-shifted as compared to those of the corresponding complexes with individual N-oxides) [8]. TD DFT calculations indicated the presence of equivalent low-energy absorption bands for the complexes of protonated and Zn(II)—coordinated NQO (Table S1 in the Supporting Information). However, the presence of uncoordinated N-oxides in the solution with the highest concentrations of ZnCl_2_ together with the low solubility of pyrene in solvents suitable for the dissolution of zinc chloride precluded a quantitative UV-Vis spectral measurements of the interaction of Zn-coordinated N-oxides with pyrene. As such, the new absorption band of such complexes was observed only in the solutions of protonated NQO (Figure S4 in the Supporting Information). Overall, the liquid-phase studies indicated that despite the presence of an electron-withdrawing substituent, NPQ and NQO form complexes with ZnCl_2_, and the presence of the latter affects the interaction between N-oxides and pyrene in a similar way as boron trifluoride. 

### 2.2. X-ray Structural Studies of the Associations of the Zn-Coordinated N-Oxides with Pyrene 

**Figure 2:**
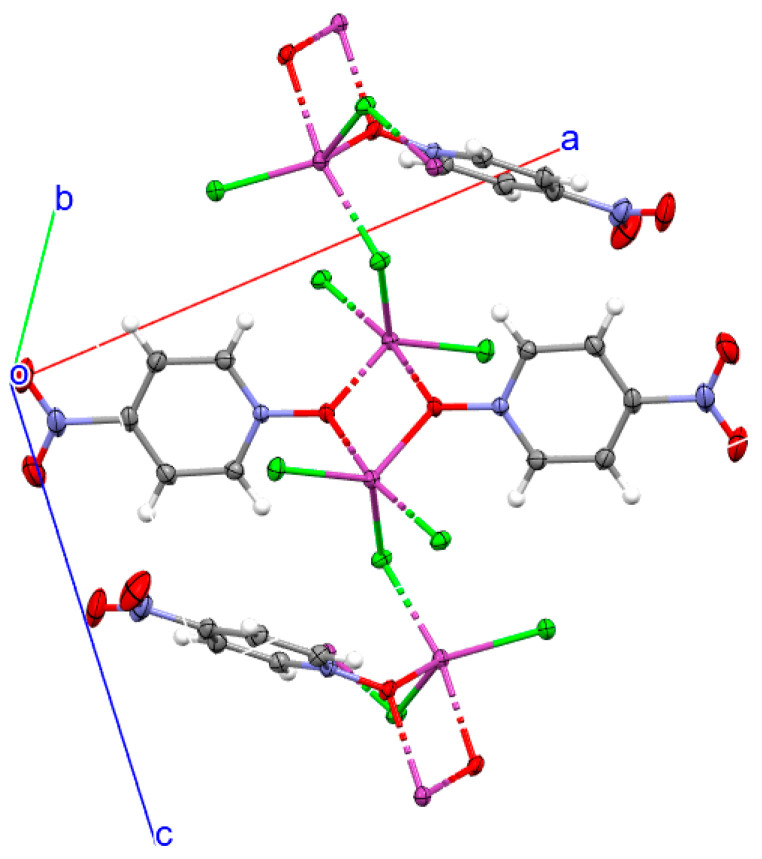
Fragment of the X-ray structure of ZnCl_2_(NPO) (**1**) comprising Zn-coordinated NPO. Dash-dotted lines show longer Zn-O and Zn-Cl bonds. Color code: dark gray—carbon, light gray—hydrogen, red—oxygen, blue—nitrogen, magenta—zinc, and green—chlorine.

Mixing zinc(II) chloride and NPO in acetone (in a 10:1 molar ratio) resulted in the formation of essentially colorless crystals (**1**) comprising ZnCl_2_ and N-oxide in a 1:1 ratio. Each zinc(II) ion coordinates two oxygen atoms from two NPO molecules. In turn, each oxygen is coordinated to two zinc(II) ions. Such bonding produced centrosymmetric 2:2 adducts forming 4-membered rings (Figure 2). They comprised pairs of two non-equivalent Zn-O bonds of 2.002 Å and 2.348 Å (the latter is shown as a dash-dotted line in Figure 2). Besides the coordination of two oxygens, each zinc(II) ion coordinates three Cl^−^ anions. Two of them are shared by two zinc ions. The Zn-Cl bond lengths with these bridging chlorides (2.403 Å) are noticeably higher than that of chloride coordinated with just one zinc (2.215 Å). Overall, zinc(II) is characterized by distorted trigonal–bipyramidal geometry which is rather rare for these ions. 

**Figure 3:**
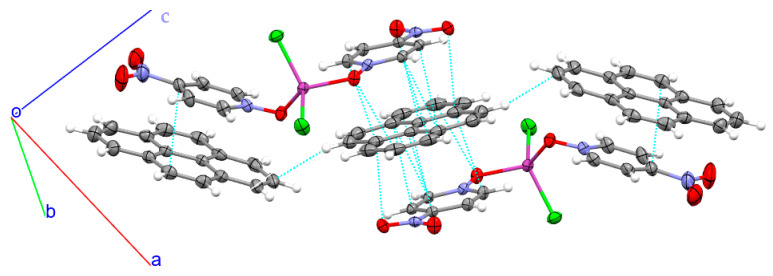
Fragment of the X-ray structure of co-crystals [(ZnCl_2_(NPO)_2_] _2_(pyrene)_5_ (**2**) comprising Zn-coordinated NPO with pyrene showing binary and ternary donor–acceptor complexes. Light blue lines show contacts shorter than the van der Waals separations. Color code: dark gray—carbon, light gray—hydrogen, red—oxygen, blue—nitrogen, magenta—zinc, and green—chlorine.

Crystallization from solutions containing ZnCl_2_ and NPO (in 1:2 molar ratio) together with a large excess of pyrene resulted in the formation of red triclinic crystals of [ZnCl_2_(NPO)_2_]_2_(pyrene)_5_ (**2**). They comprised zinc ions coordinated to two Cl^−^ anions and two NPO molecules. Notably, such coordination of two N-oxides to zinc(II) halides and the tetrahedral geometry of the [ZnCl_2_(NPO)_2_] complex represents a common motif in the associations of zinc(II) halides with various N-oxides [24,25,26,27], as opposed to the rare 2:2 Zn:NPO binding and trigonal–bipyramidal geometry of zinc complexes in **1.** The most important for the current work was the fact that crystal **2** contains pyrene in a 2:5 zinc–pyrene ratio. Two NPO molecules from two [ZnCl_2_(NPO)_2_] complexes form *π*-stacked ternary units with one pyrene molecule in the center. This ternary complex shows multiple contacts between the essentially co-planar NPO and pyrene molecules (Figure 3 and Figure S5 in the Supporting Information). The remaining NPO molecules from the two zinc complexes are located on the opposite sides of the ternary complex. Each of these N-oxides forms 1:1 complexes with pyrene. However, in contrast to the ternary associations, there is only one contact shorter than the van der Waals separation in each of these *π*-stacked dyads. These stacks are separated by pyrene molecules which do not interact with the N-oxides (Figure S5 in the Supporting Information). 

**Figure 4:**
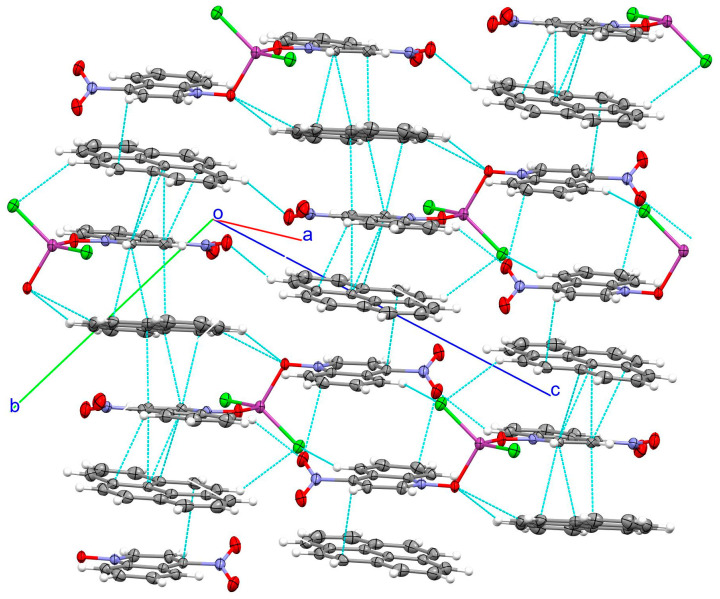
Fragment of the X-ray structure of co-crystals [(NQO)_2_ZnCl_2_](pyrene)_3_ (**3**) comprising Zn-coordinated NQO with pyrene showing heptameric donor–acceptor associations. Light blue lines show contacts shorter than the van der Waals separations. Color code: dark gray—carbon, light gray—hydrogen, red—oxygen, blue—nitrogen, and green—chlorine.

Crystallization from the solutions of ZnCl_2_ with another N-oxide, NQO, and an excess of pyrene resulted in the formation of also red co-crystals of [(NQO)_2_ZnCl_2_]_2_(pyrene)_3_ (**3**) comprising *π*–*π* bonded associations between the pyrene and zinc-coordinated N-oxide molecules (Figure 4). These associations represent the centrosymmetric heptameric complexes consisting of alternating (and essentially co-planar) four N-oxides and three pyrene molecules. Each pyrene molecule in the heptamer forms one or several contacts (on both sides) with NQO molecules that are shorter than van der Waals separation. The terminal NQO molecules in one heptamer neighbor similar molecules in the other heptamers with two short contacts between oxygens in the nitro groups of one NQO and with a carbon atom in the quinoline core of its neighbor. 

**Figure 5:**
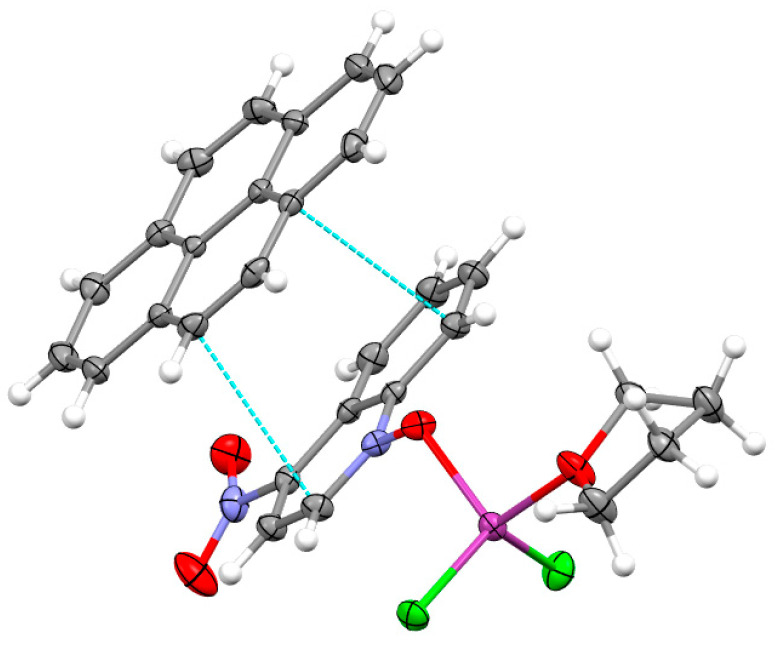
Fragment of the X-ray structure of co-crystals [ZnCl_2_(THF)(NQO)]_2_(pyrene)_3_ (**4**) containing Zn-coordinated NQO with pyrene showing 1:1 complex between N-oxide and pyrene (view along an *a* axis). Light blue lines show contacts shorter than the van der Waals separations. Disorder for the THF molecule is omitted for clarity. Color code: dark gray—carbon, light gray—hydrogen, red—oxygen, blue—nitrogen, magenta—zinc, and green—chlorine.

Crystallization of the similar mixtures of ZnCl_2_, NQO, and pyrene from solutions containing THF produced dark red (nearly black) crystals of [ZnCl_2_(THF)(NQO)]_2_(pyrene)_3_ (**4**). Each zinc ion is coordinated with one NQO and one solvent molecule in these co-crystals (Figure 5). Each N-oxide in turn forms a 1: 1 complex with pyrene. Two contacts between essentially co-planar donor and acceptor (with an angle of 5.4° between the planes of these molecules) were shorter than the van der Waals separations. The [ZnCl_2_(THF)(NQO)]pyrene associations are separated by another (non-bonded) pyrene. 

**Figure 6:**
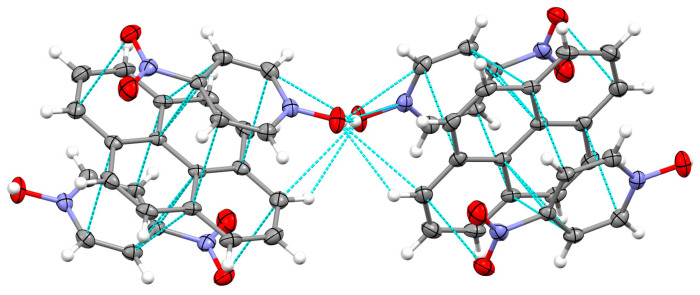
Fragment of the X-ray structure (view along an a axis) of co-crystals (I_9_^−^)(NPO-H^+^-NPO)(pyrene) (**5**) containing hydrogen-bonded pairs of NPO and pyrene (the halogen-bonded nonaiodide network is omitted for clarity; it is shown in Figure S6 in the Supporting Information). Light blue lines show contacts shorter than the van der Waals separations. Color code: dark gray—carbon, light gray—hydrogen, red—oxygen, and blue—nitrogen.

To compare the effects of coordination to metal ions with those resulting from halogen bonding, we also tried to prepare the associations of pyrene with halogen-bonded N-oxide molecules. However, crystallization from solutions containing pyrene and NPO together with a strong halogen-bond donor, diiodine, produced co-crystals (I_9_^−^)(NPO-H^+^-NPO)(pyrene) (**5**) containing pairs of hydrogen-bonded N-oxide molecules (Figure 6). 

The proton that links two NPO molecules is disordered between two positions (i.e., between bonding to one oxygen and hydrogen bonding to another, and vice versa; note that the crystallographic database contains several similar hydrogen-bonded pairs of N-oxide molecules [29,30,31]). The charge of the proton in these crystals is balanced by an iodide anion which forms two stronger and two weaker halogen bonds with four diiodine molecules (with halogen bond lengths of 3.181 Å, 3.451 Å, and 3. 518 Å, see Figure S6 in the Supporting Information). These co-crystals also contain pyrene molecules. Each pyrene is sandwiched between two NPO molecules. These ternary associations comprise multiple contacts between pyrene and NPO that are shorter than the van der Waals separations. 

The selected geometric characteristics of the complexes of N-oxides with ZnCl_2_ and pyrene are listed in Table 1. For comparison, this table also contains the related characteristics of the complexes with the protonated N-oxides as well as reported earlier ternary complexes with boron trifluoride. 

**Figure 7:**
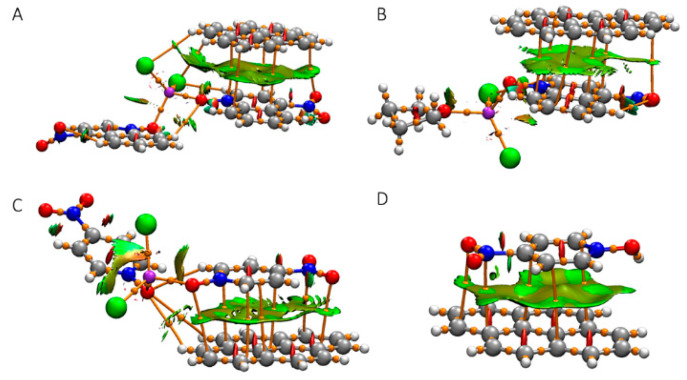
Superposition of the results of the QTAIM and NCI analyses onto the structure of the *π*–*π* bonded complexes between N-oxides and pyrene extracted from the X-ray structures of co-crystals [(NQO)_2_ZnCl_2_]_2_(pyrene)_3_ (**3**) (**A**), [ZnCl_2_(THF)(NQO)]_2_(pyrene)_3_ (**4**) (**B**), [ZnCl_2_(NPO)_2_]_2_(pyrene)_5_ (**2**) (**C**), and I_9_^−^)(NPO-H^+^-NPO)(pyrene) (**5**) (**D**); note that only one N-oxide is shown and polyiodide is omitted for clarity). The bond paths and critical (3, −1) points (from QTAIM) are shown as orange lines and spheres, and blue–green areas (from NCI) indicate bonding interactions. Atom color code: dark gray—carbon, light gray—hydrogen, red—oxygen, blue—nitrogen, magenta—zinc, and green—chlorine.

The data in Table 1 show that the Zn-O bond lengths in the associations with pyrene are close to those reported earlier in the complexes of zinc(II) with other N-oxides (about 2.00 Å [24,25,26,27]) and a shorter Zn-O bond in crystal **1**. Coordination with Zn(II) resulted in the increase in the N-O bond lengths in the N-oxides to around 1.33 Å, as compared to about 1.29 Å reported for the individual molecules [34,35]. The C-N bonds in the Lewis acid-bonded N-oxides were also elongated to 1.47–1.48 Å as compared to 1.46 Å measured in the individual molecules. The N-O distance in the (partially) protonated NPO was slightly longer than that in the complexes with zinc, and complexes with BF_3_ showed even longer N-O and C-N bonds. Most notably, all the N-oxide molecules in all of these co-crystals formed *π*-stacked 1:1 or higher order associations with pyrene with short contacts between the essentially co-planar molecules. This suggests a substantial bonding between pyrene and the Lewis acid-coordinated N-oxides. To confirm the presence of such bonding, we carried out the quantum theory of atoms in molecules (QTAIM) and non-covalent interaction (NCI) analyses of these co-crystals [36,37,38]. The QTAIM analyses revealed that all the associations comprise multiple bond paths with the corresponding bond critical points between the N-oxide and pyrene molecules (Figure 7). 

The NCI analyses also showed green–blue areas around each BCP indicating relatively weak attractive interactions. This indicates multicenter bonding between the N-oxides and pyrenes in these associations. To evaluate the strength of these interactions and the effects of zinc (and other Lewis acids) on these interactions, we carried out a computational analysis of these bondings as follows. 

### 2.3. Computational Analysis of Complexes of Pyrene with N-oxides

**Figure 8:**
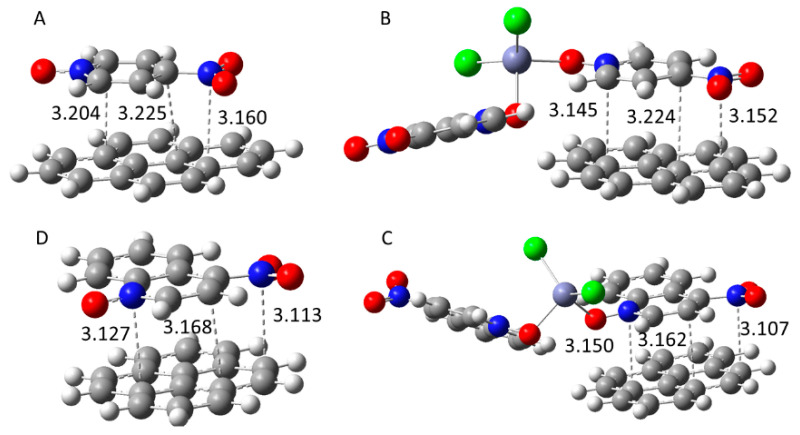
Optimized structures of the complexes between individual or Zn(II)-coordinated N-oxide with pyrene: NPO·pyrene (**A**), ZnCl_2_(NPO)_2_·pyrene (**B**), NQO·Pyrene (**C**), and ZnCl_2_(NQO)_2_·pyrene (**D**). The numbers show the interatomic distances (in Å). Atom color code: dark gray—carbon, light gray—hydrogen, red—oxygen, blue—nitrogen, magenta—zinc, and green—chlorine. Dashed lines show short intermolecular contacts and the numbers show distances (in Å).

The structures of the individual donor–acceptor associations produced by the optimization of the Zn(II)- or BF_3_-coordinated or protonated N-oxides and their complexes with pyrene are illustrated in Figure 8. 

Overall, the structural features of these complexes are consistent with the results of the X-ray structural analysis. In particular, the Zn-O distances in the optimized complexes are essentially the same (about 2.01 Å) as in the solid-state structures. Also, the coordination (or protonation) led to a substantial elongation of the N-O bonds in the N-oxides (Table 2). Most important for the current work, however, were the effects of the interaction with the Lewis acids on the electronic structures of the N-oxides and their interaction with pyrene.

All the optimized complexes showed *π*-stacked N-oxides and pyrene moieties with multiple interatomic contacts which were shorter than the sums of the van der Waals radii. Notably, these distances in the complexes of pyrene with Zn-coordinated N-oxides were slightly shorter than those in the associations involving non-coordinated acceptors. This indicates that coordination with zinc leads to a stronger attraction between N-oxides and pyrene. These structural indications were confirmed by the comparison of the binding energies, ΔE_b_. The ΔE_b_ values listed in the last column in Table 2 reveal that the coordination of N-oxides with zinc increases the stability of their *π*–*π* bonded complexes. The binding of N-oxides with BF_3_ or their protonation led to similar effects. In all the cases, the magnitudes of the binding energies are higher than those found for the individual N-oxide molecules. The free energies of the formation of the complexes with pyrene, ΔG, show similar trends. However, due to the entropy loss during complex formation, these values are much less negative. 

### 2.4. Origin of the Lewis Acid Effects on the Multicenter Donor–Acceptor Bonding 

The classic Mulliken theory explained the formation of the complexes, such as that between pyrene and N-oxide, in terms of the mixing of the highest occupied molecular orbital (HOMO) of the electron donor with the lowest unoccupied orbital of the acceptor (LUMO), resulting in partial charge transfer [39]. The stability of such complexes is determined, in part, by the HOMO/LUMO gap of the interacting species. As such, an increase in its electron-acceptor strength (i.e., the lowering of the LUMO energy of the acceptor) in the complexes with the same donor results in more stable complexes. In comparison, the *σ*- and *π*-hole concepts which were developed during the last two decades relate the formation of the supramolecular complexes to the electrostatic attraction of electron-rich species to the areas of the positive potential on the surfaces of electron acceptors [40]. However, while the *σ*- and *π*-hole concepts were extensively utilized in the analysis of the recently recognized types of supramolecular interactions (e.g., halogen, chalcogen, or anion-*π* bondings), their applications for the *π*-stacked donor–acceptor complexes are scarce. 

**Figure 9:**
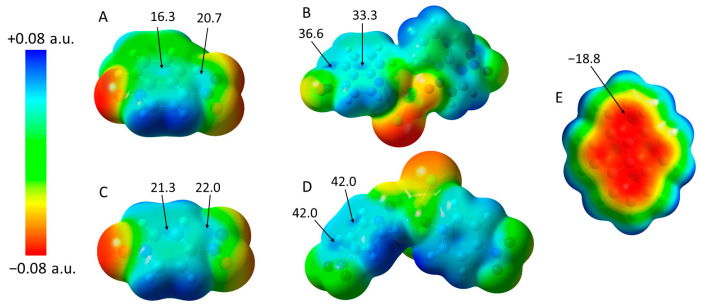
Electrostatic potential (calculated at 0.001 electron bohr^−^^3^ electronic density) on the molecular surfaces of NQO (**A**), ZnCl_2_(NQO)_2_ (**B**), NPO (**C**), ZnCl_2_(NPO)_2_ (**D**), and pyrene (**E**). The numbers show the ESP values (in kcal/mol) at *π*-holes over the molecular frameworks of N-oxides (in **A**–**D**) and the most negative potential on the surface of pyrene.

The maps of the surface electrostatic potentials of pyrene and individual and Lewis acid-coordinated N-oxides are shown in Figure 9 and Figure S7 in the Supporting Information. These maps revealed that the area over the aromatic core of the pyrene is characterized by negative potentials, and its side (the area along the extension of the C-H bond) shows positive potential. 

The most positive potentials on the surface of N-oxides are also located over their hydrogen substituents. However, N-oxides show two areas of positive potential over their molecular framework, i.e., *π*-holes, above the pyridinium ring center and C-NO_2_ fragment. The latter potential is somewhat larger in the individual NPO and NQO molecules (Table 2), and the coordination of NPO or NQO to Zn(II) increases the magnitude of both of these *π*-holes (similar to the enhancement of the *π*-holes over the C-NO_2_ fragment of NPO resulting from the halogen or hydrogen bonding of this N-oxide or its coordination to BF_3_ reported by Frontera et al. [15]). Thus, the increase in the magnitude of ΔE in the complexes with coordinated or protonated N-oxides as compared to that in the individual acceptors can be related to the increase in the potential of the *π*-holes. This implies that the electrostatic attraction between the areas of the negative potentials on the surface of pyrene with the areas of the positive potential over the pyridinium ring and the C-NO_2_ fragments of N-oxides plays a significant role in the formation of the *π*-stacked complexes. However, the correlation between ΔE and V*_π_*_holes_ is rather weak (they are characterized by the R^2^ values of 0.67 and 0.77 for the complexes with NQO and NPO, respectively, and 0.53 if all the complexes are considered in a single correlation; see Figure S8 in the Supporting Information). Also, the binding of pyrene with NQO is stronger than that with NPO, even though the V*_π_*_hole_ values on the surface of the latter are higher. Apparently, besides electrostatics, other factors contribute to the binding between N-oxides and pyrene. Since the NQO surface is larger, dispersion probably represents one of the factors leading to the stronger binding of this molecule with pyrene. Another factor could be related to the molecular orbital interactions. Specifically, the energy of LUMO of NQO is lower. According to the Mulliken theory [39], this leads to higher donor–acceptor charge transfer and stronger binding. Natural Bond Orbital (NBO) analysis confirmed that charge transfer from pyrene to NQO is slightly larger than to NPO. Furthermore, coordination to zinc, BF_3_, or protonation substantially decreases the energies of the LUMOs of NPO and NQO, and the correlation between the energies of LUMO and ΔE (with R^2^ = 0.68 for all the complexes; see Figure S8 in the Supporting Information) is somewhat better than that between ΔE and V*_π_*_hole_ (it should be mentioned, though, that the variation in the LUMOs’ energies is correlated with the values of V*_π_*_holes_, which hinders the separation of the effects of these two factors). Moreover, earlier studies showed that the interaction of various aromatic donors (including pyrene) with BF_3_-coordinated NQO molecules led to the appearance of a new absorption band of the complex that followed the Mulliken correlation [8]. These observations suggest the significant contribution of molecular–orbital (charge-transfer) interactions in the formation of these complexes. 

To quantify the contributions of the electrostatic and molecular–orbital interactions, as well as dispersion to the bonding between N-oxides and pyrene, we carried out an energy-decomposition analysis (EDA) of interaction energies in these associations. In particular, the EDA analysis using the AMS suite of programs decomposes the intermolecular interaction energy values into its electrostatic, ΔE_elstat_; Pauli repulsion, ΔE_Pauli_; orbital (charge-transfer) interaction, ΔE_oi_; and dispersion, ΔE_disp_ components: [41,42]

ΔE_int_ = ΔE_elstat_ + ΔE_Pauli_ + ΔE_oi_ + ΔE_disp_ (1)

The values of the interaction energies and their components for the complexes of pyrene with Zn(II)- or BF_3_-coordinated and individual N-oxides are listed in Table 3.

The data in Table 3 show that coordination to ZnCl_2_ or BF_3_ increases all three components of the attractive interaction. These increases are compensated, to some extent, by the increase in the Pauli repulsion (due to the closer approach of the interacting molecules). As such, none of the individual attractive components (i.e., ΔE_elstat_, ΔE_oi_, or ΔE_disp_) would be sufficient, if taken separately, to account for the increase in the magnitude of the interaction energy. 

In conclusion, our results indicate that the effects of the Lewis acid on the multicenter donor–acceptor bonding are related both to the enhancement of the *π*-holes on the surface of the N-oxides and the strengthening of their electron-acceptor properties (as reflected in the lower energies of their LUMOs). These factors lead to the increase in the magnitudes of the (attractive) electrostatic and orbital interactions, which bring donor and acceptor closer together and further enhance dispersion interaction as well as Pauli repulsion. The combination of all these components produces a stronger *π*–*π* binding of N-oxides and a model *π*-donor, pyrene. 

## 3. Materials and Methods

Commercially available ZnCl_2_, NQO, and pyridine-N-oxide (all from Sigma Aldrich, St. Louis, MO, USA), as well as NPO and 4-methoxypyridine-N-oxide (all from TCI, Tokyo, Japan) were used without additional purification. The UV-Vis measurements were carried out on a Cary 5000 spectrophotometer (Agilent, Santa Clara, CA, USA) as described before [8,14].

Single crystals **1** of the Zn(II)-coordinated NPO were prepared by the dissolution of N-oxide in acetone and adding a 10-fold excess of ZnCl_2_. After the removal of the remaining undissolved material (by centrifuging), the solution was placed in a refrigerator (4 °C). Essentially colorless crystals **1** containing ZnCl_2_ and NPO were formed after 24 h. Co-crystals containing Zn-coordinated nitropyridine-N-oxide and pyrene were prepared by mixing ZnCl_2_ and NPO in a 1:2 molar ratio together with a ten-fold excess of pyrene in dichloromethane. This mixture was cooled down to −20 °C and kept at this temperature for several weeks. During this time, red crystals of [ZnCl_2_(NPO)_2_]_2_(pyrene)_5_ (**2**) were formed. A similar procedure with NQO instead of NPO produced crystals [ZnCl_2_(NQO)_2_]_2_(pyrene)_3_ (**3**). The crystallization of analogous three-components mixture from dichloromethane–tetrahydrofuran (1:1) solution produced [ZnCl_2_(THF)(NQO)]_2_(pyrene)_3_ crystals (**4**). Crystals **5** were prepared by mixing NPO, pyrene, and diiodine in a 1:1:1 molar ratio in dichloromethane. This mixture was cooled down to −30 °C and kept at this temperature in an open vial for several weeks. This resulted in the formation of dark crystals **5**, (I_9_^−^)(NPO-H^+^-NPO)(pyrene). Note that due to the limited solubility of N-oxides and pyrene, the crystals of the complexes were obtained as mixtures with the crystals of the individual components. Also, multiple attempts to prepare single crystals of ZnCl_2_(NQO) (using various solvents and conditions) resulted in the precipitation of the individual NQO. This is related to the limited solubility of NQO in the solvents suitable for the dissolution of ZnCl_2_ and the low formation constants of the complex formation. The formation of the ternary complexes in the presence of pyrene indicates (in agreement with [15]) that bonding with pyrene facilitates coordination to zinc, and vice versa. 

The single crystal structures were determined on a Bruker Quest diffractometer (Bruker AXS, LLC, Madison, WI, USA) with a fixed chi angle, a sealed tube fine focus X-ray tube, a single crystal curved graphite incident beam monochromator, a PhotonII area detector (Bruker AXS, LLC, Madison, WI, USA), and an Oxford Cryosystems low temperature device (Oxford Cryosystems, Oxford OX29 8LH, United Kingdom). The examination and data collection were performed with Mo Kα radiation (λ = 0.71073 Å). The reflections were indexed and processed, and the files were scaled and corrected for absorption using APEX4 [43]. The space groups were assigned using XPREP within the SHELXTL suite of programs, and the structures were solved by dual methods using ShelXT and refined by full-matrix least-squares against F^2^ with all the reflections using the graphical interface Shelxle [44,45,46,47]. If not specified otherwise, the H atoms attached to carbon atoms were positioned geometrically and constrained to ride on their parent atoms, with the C-H bond distances of 1.00, 0.99, and 0.98 Å for aliphatic CH_2_ moieties, respectively. The methyl H atoms were allowed to rotate but not to tip to best fit the experimental electron density. The U_iso_(H) values were set to a multiple of U_eq_(C) with 1.5 for CH_3_ and 1.2 for C-H units, respectively. Crystallographic, data collection, and refinement details are listed in Table S2 in the Supplementary Materials. Complete crystallographic data, in CIF format, have been deposited with the Cambridge Crystallographic Data Centre. CCDC 2358284–2358287 and 2365181 contain the supplementary crystallographic data for this paper. These data can be obtained free of charge via www.ccdc.cam.ac.uk/data_request/cif. 

The geometries of the complexes and their components were optimized without constraints via M06-2X/6-31G* calculations (in dichloromethane, with a polarizable continuum model) using the Gaussian 09 suite of programs [48,49,50]. The absence of imaginary frequencies confirmed that the optimized structures represent true minima. The binding energies were determined as follows: ΔE = E_comp_ − (E_Nox_ + E_pyr_), where E_comp_, E_Nox_, and E_pyr_ are the sums of the electronic and zero-point vibrational energy of the complex, pyrene, and N-oxide containing fragment (i.e., either protonated, Zn- or BF_3_-coordinated or individual N-oxide). Since the formation of the complex is accompanied by the distortion of the reactants, the binding energy represents a combination of preparation (distortion) energy, E_prep_, and interaction energy between distorted fragments, ΔE_int_. The calculated UV-Vis spectra of the complexes (Figure S9 in the Supporting Information) were obtained via TD-DFT computations (using TD(NStates = 10) keyword) at the same level. QTAIM and NCI analyses [36,37,38] were performed with Multiwfn [51] using wfn files generated by Gaussian 09. The results were visualized using the molecular graphics program VMD [52]. The details of the calculations, energies, geometric and spectral characteristics, as well as the atomic coordinates of the calculated complexes are listed in the ESI. The energy decomposition analyses (EDA) were carried out using the Amsterdam Density Functional (ADF) of the Amsterdam Modelling Suite [41,42] via single-point calculations with B3LYP-D3 functional (since it allowed the evaluation of the dispersion component of the interaction energy) and the TZ2P basis set available in AMS using the atomic coordinates of the complexes optimized as described above. 

## Figures and Tables

**Table 1:** Selected distances (in Å) in the co-crystals of N-oxides with pyrene.

Co-Crystal	M-O ^a^	N-O ^b^	C-N ^c^	C-C ^d^
ZnCl_2_(NPO) (**1**)	2.0025 (12)	1.3360 (14)	1.473 (2)	N/A
	2.348 (1)			
[ZnCl_2_(NPO)_2_]_2_(pyrene)_5_ (**2**)	2.0293 (9)	1.3311 (13)	1.4682 (18)	3.3849 (17)
	1.9903 (9)	1.3236 (12)	1.4657 (13)	3.3024 (16)
[(NQO)_2_ZnCl_2_]_2_(pyrene)_3_ (**3**)	2.0186 (12)	1.3158 (18)	1.477 (2)	3.1957 (19)
	2.0122 (13)	1.3354 (19)	1.479 (2)	3.2439 (17)
[ZnCl_2_(THF)(NQO)]_2_(pyrene)_3_ (**4**)	2.0214 (9)	1.3302 (11)	1.4773 (14 )	3.3448 (15)
(I_9_^−^)(NPO-H^+^-NPO)(pyrene) (**5**)	N/A	1.344 (4)	1.473 (5)	3.318 (6)
[BF_3_-NQO](pyrene) ^e^	1.516	1.358	1.492	3.260
[BF_3_-NPO](pyrene) ^e^	1.518	1.375	1.483	3.351

^a^ Zn-O or B-O distances; ^b^ N-oxide group; ^c^ aromatic carbon to nitrogen of nitro group; ^d^ shortest interatomic distance between N-oxide and pyrene; ^e^ from [8].

**Table 2:** Selected characteristics of the optimized individual or Lewis acid-bonded N-oxides and their complexes with pyrene.

N-Oxide	V_πhole_, kcal/mol ^a^	E_LUMO_, ^b^ eV	d_N-O_, ^c^ Å	ΔE_b_, ^d^ kcal/mol	ΔG, ^e^ kcal/mol	Δq, ^f^ e
NPO	21.3/22.0	−1.88	1.264 (1.266)	−11.1	1.69	0.008
NPO-H^+^	128.6/109.8	−3.40	1.366 (1.366)	−16.4	−3.20	0.053
NPO-BF_3_	48.9/48.3	−2.66	1.340 (1.341)	−13.6	−0.01	0.022
ZnCl_2_(NPO)_2_	42.0/42.0	−2.44	1.314 (1.311)	−14.5	0.47	0.015
NQO	16.3/20.7	−2.06	1.264 (1.264)	−14.1	−0.45	0.010
NQO-H	116.7/105.4	−3.47	1.367 (1.368)	−15.9	−2.89	0.063
NQO-BF_3_	40.2/45.2	−2.73	1.342 (1.333)	−14.2	−0.35	0.024
ZnCl_2_(NQO)_2_	33.3/36.6	−2.54	1.315 (1.312)	−15.3	−1.69	0.023

^a^ Electrostatic potentials over the pyridinium ring and the C-NO_2_ fragment (vide infra); ^b^ Energy of the LUMOs; ^c^ N-O bond lengths in the individual or Lewis acid-bonded N-oxide. The values in parenthesis show the bond length in the corresponding complexes with pyrene; ^d^ Binding energies between pyrene and (individual or coordinated) N-oxide; ^e^ Free energy change in complex formation; ^f^ Charge transfer from pyrene.

**Table 3:** Component of interaction energy (in kcal/mol) in the complexes of N-oxides with pyrene (from the EDA analysis).

Complex ^a^	ΔE_elstat_	ΔE_oi_	ΔE_disp_	ΔE_Pauli_	ΔE_int_
NPO·pyrene	−11.3	−5.1	−17.1	23.2	−10.3
BF_3_-NPO·pyrene	−15.8	−7.2	−18.3	26.1	−15.2
ZnCl_2_(NPO)_2_·pyrene	−16.3	−7.6	−22.4	30.4	−15.9
NQO·pyrene	−15.6	−5.9	−22.0	30.7	−12.8
BF_3_-NQO·pyrene	−18.5	−8.0	−23.8	33.6	−16.8
ZnCl_2_(NQO)_2_·pyrene	−16.6	−7.1	−25.8	32.8	−16.7

^a^ note that values for the positively charged complex with the protonated N-oxides showed similar trends, but only data for the neutral complexes are shown for consistent comparison.

## Data Availability

Data are contained within the article and Supplementary Materials.

## Supplementary Materials

The following supporting information can be downloaded at: https://www.mdpi.com/article/10.3390/molecules29143305/s1. Figure S1: UV-Vis spectral changes upon the addition of ZnCl_2_ to the solutions of 4-methoxypyridine-N-oxide and NQO. Figure S2: UV-Vis spectral changes upon the addition of HPF_6_ to the solutions of NPO and NQO. Figure 3: Color changes upon formation of complexes with pyrene. Figure S4: UV-Vis spectra of the dichloromethane solutions of NQO, pyrene, and their complexes. Figure S5: X-ray structure of co-crystals **1**. Figure S6: Halogen-bonded networks formed by I_2_ and I^−^ in crystals **4**. Figure S7: Electrostatic potential maps. Figure S8: Correlations between binding energy between N-oxides and pyrene and average values of the potentials of the π-holes on the surfaces of N-oxides (left) or energies of their LUMOs. Figure S9: Calculated spectra of the complexes. Table S1: Energies of the HOMO and LUMO of the individual and bonded to Lewis acid N-oxides and the absorption maxima of their (calculated and experimentally measured) charge-transfer bands with pyrene. Table S2. Selected characteristics of the optimized individual or Lewis acid-bonded N-oxides and their complexes with pyrene. Table S3. Free energy of individual or Lewis-acid bonded N-oxides and their complexes with pyrene. Table S4. Crystallographic, data collection, and refinement details. Atomic coordinates of individual and Lewis acid-bonded N-oxides and their complexes with pyrene.
