# Supplementary material for: Complexes of Zinc-Coordinated Heteroaromatic N-Oxides with Pyrene: Lewis Acid Effects on the Multicenter Donor–Acceptor Bonding
Source: Molecules. 2024 Jul 13;29(14):3305. doi: 10.3390/molecules29143305 (PMC11279733; doi:10.3390/molecules29143305)
Supplement: Supplementary file 1 [file molecules-29-03305-s001.zip › molecules-3105148-supplementary.pdf]

Supporting Information

available for

Complexes of Zinc-coordinated heteroaromatic N-oxides with pyrene: Lewis acids effects on the multicenter donor/acceptor bonding

Yakov P. Nizhnik,[1] Erin Hansen,[2] Cayden Howard,[2] Matthias Zeller[3] and Sergiy V. Rosokha[2]*

[1] *BioStone, 2815 Exchange Blvd., Southlake, TX, 76092, United States*
[2] *Department of Chemistry, Ball State University, Muncie, Indiana, 47306, United States;*
[3] *Department of Chemistry, Purdue University, West Lafayette, IN, United States*
* Correspondence: svrosokha@bsu.edu

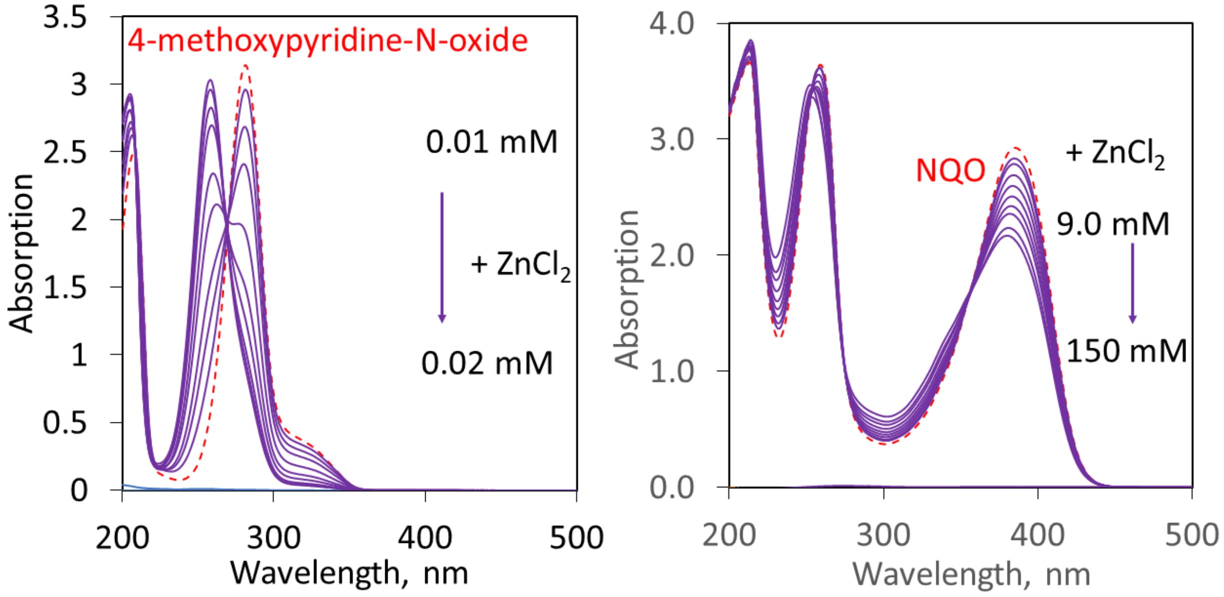

Figure S1. Spectra of the dichloromethane solutions with a constant concentration of 4-methoxypyridine-N-oxide (left) and NQO (right) and various concentrations of $ZnCl_2$. Spectra of the solutions of individual N-oxides are shown as dashed red lines (absorption of $ZnCl_2$ is negligible in this spectral range).

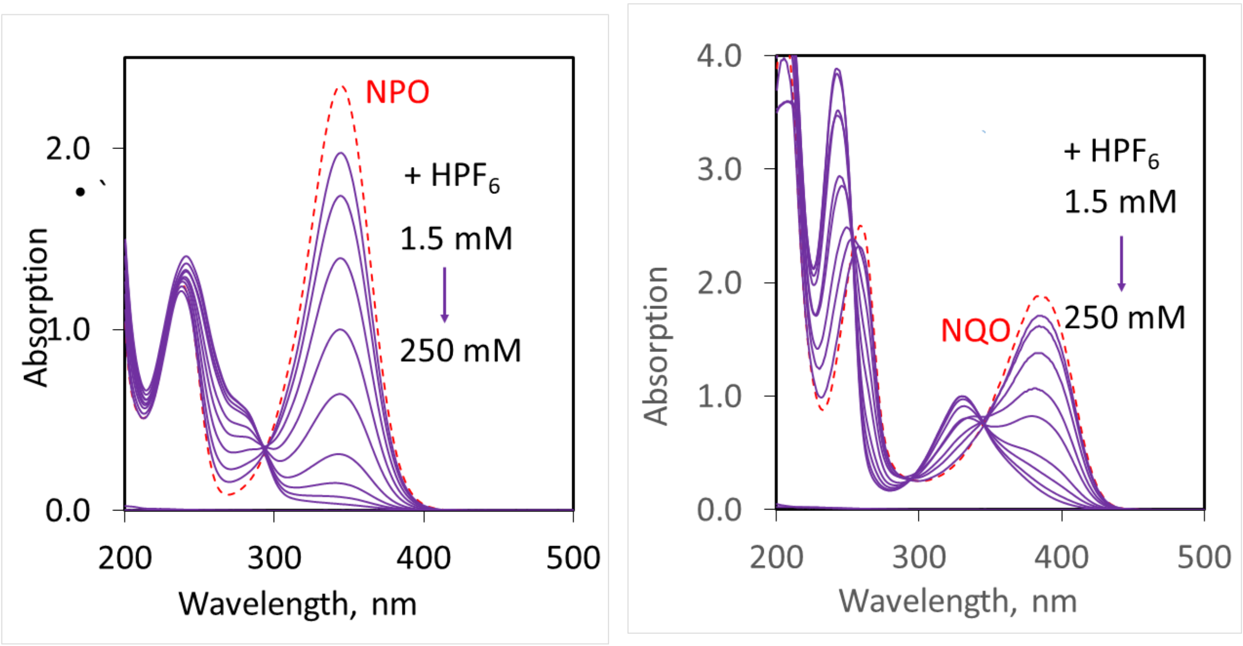

Figure S2. Spectra of the dichloromethane solutions with a constant concentration of NPO (left) and NQO (right) and various concentrations of HPF_6. Spectra of the solutions of individual N-oxides are shown as dashed red lines (absorption of HPF_6 is negligible in this spectral range).

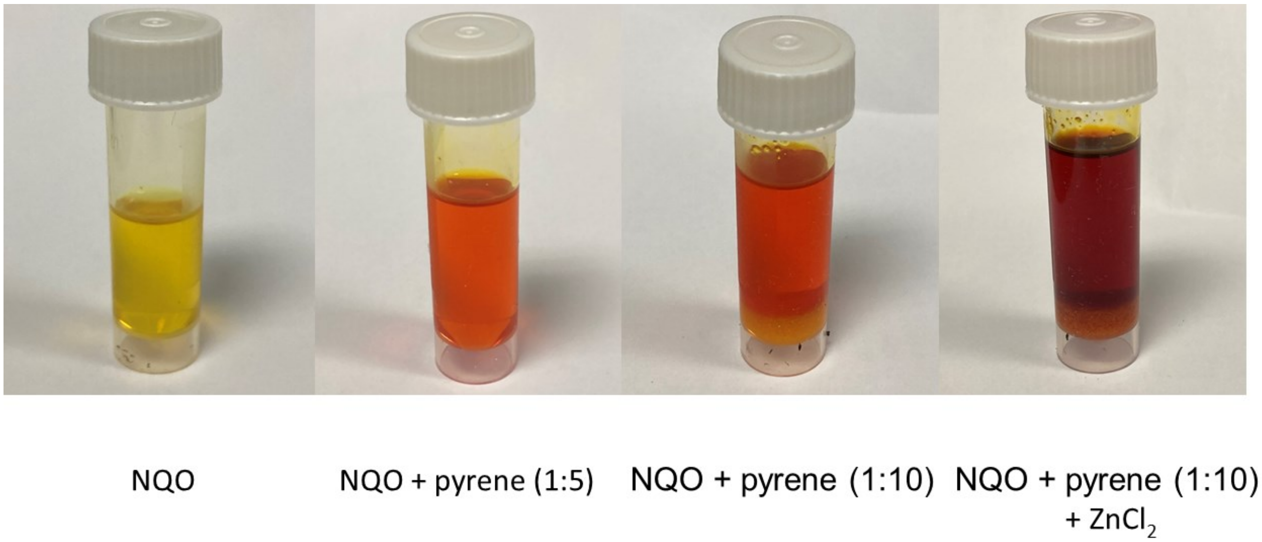

Figure S3. Color changes resulting from the interaction of NQO and pyrene in the absence and in the presence of $ZnCl_2$

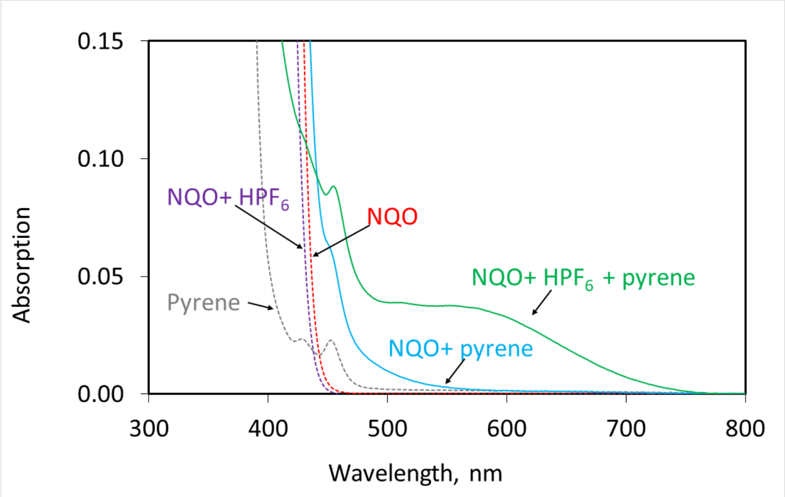

Figure S4. Spectra of the dichloromethane solutions of NQO (right), pyrene, and their complexes (as indicated).

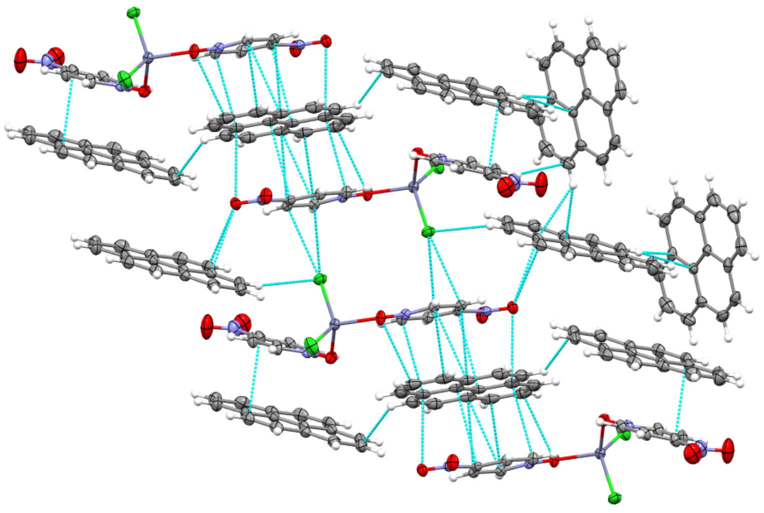

Figure S5. Fragment of the X-ray structure of co-crystals **1** comprising Zn-coordinated NPO with pyrene showing trimeric and dimeric donor/acceptor complexes. Light blue lines show contacts shorter than the van der Waals separations.

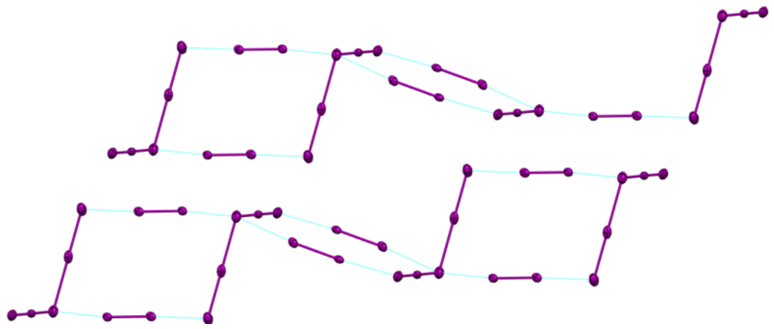

Figure S6. Fragment of the X-ray structure of co-crystals **4** showing halogen-bonded networks formed by diiodine and iodide. Light blue lines show contacts shorter than the van der Waals separations.

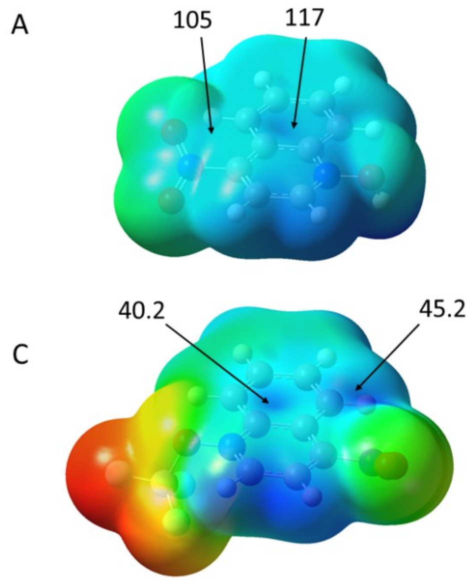

Figure S7. Electrostatic potential (calculated at 0.001 electron bohr^{-3} electronic density) on the molecular surfaces of : NQO-H$^+$ (A), NPO-H$^+$ (B), NQO·BF_3 (C), NPO·BF_3 (D). Numbers show ESP values (in kcal/mol) at π-holes over the molecular frameworks of N-oxides. (Blue color represent positive potential and red color represent negative potential).

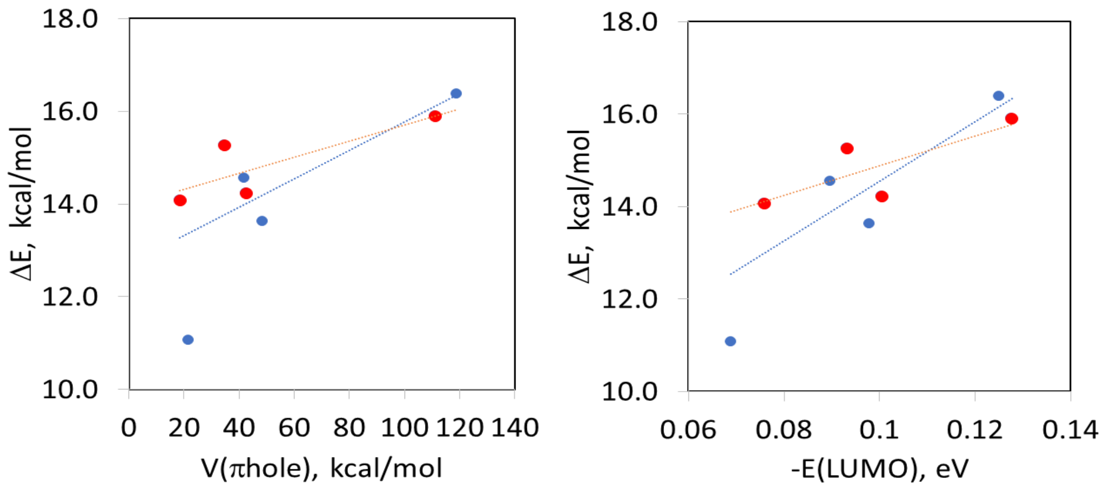

Figure S8. Relationship between binding energy between N-oxides and pyrene and average values of the potentials of the π-holes on the surfaces of N-oxides (left) or energies of their LUMOs (right). The values for NQP and NPO are shown as red and blue circles, respectively.

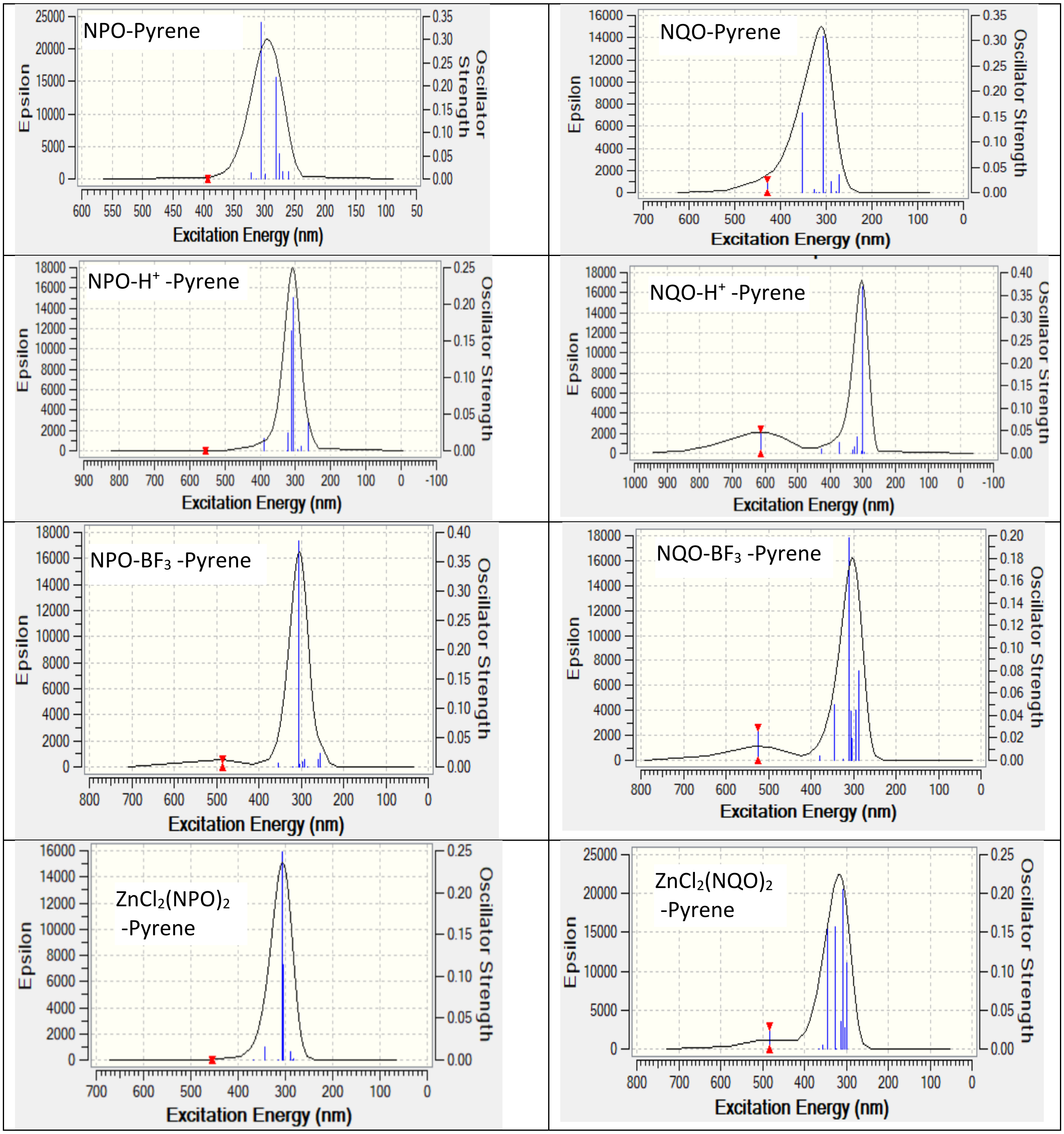

Figure S9. UV-Vis spectra of the complexes of pyrene with individual or Lewis-acid bonded N-oxides (as indicated) obtained from TD DFT calculation (TD(NStates=10) keyword).

Table S1. Energies of the HOMO and LUMO of individual and bonded to Lewis acids N-oxides and absorption maxima of their (calculated and experimentally measured) charge-transfer bands with pyrene

	E(LUMO), eV	E(HOMO), eV	λ_{max}, nm (calc)[a)]	λ_{max}, nm (exp)
NPO	-0.2953	-0.0689	-	-
NPO-H^+	-0.4109	-0.1251	389sh	-
NPO-BF3	-0.3816	-0.0978	485	500
$ZnCl_2(NPO)_2$	-0.3324	-0.0896	-	-
NQO	-0.2804	-0.0759	428sh	-
NQO-H	-0.3511	-0.1276	612	560
NQO-BF3	-0.3243	-0.1004	525	510
$ZnCl_2(NQO)_2$	-0.3085	-0.0932	483	-

a) See Figure S9 for the spectra.

Table S2. Selected characteristics of the optimized individual or Lewis-acid-bonded N-oxides and their complexes with pyrene

	Individual or Lewis-acid-bonded N-oxides			Complexes with pyrene		
	E, a.u.	ZPE, a.u.	d_{O-N}, Å	E, a.u.	ZPE, a.u.	d_{O-N}, Å
NPO	-527.73664	0.09732	1.264	-1143.27347	0.30802	1.266
NPO-H^+	-528.14960	0.10991	1.366	-1143.69507	0.32078	1.367
NPO-BF3	-852.23550	0.11240	1.340	-1467.77685	0.32354	1.341
$ZnCl_2(NPO)_2$	-3755.07924	0.19978	1.312/1.315	-4370.62193	0.41078	1.309/1.314
NQO	-681.31738	0.14491	1.264	-1296.85914	0.35574	1.264
NQO-H	-681.73204	0.15763	1.367	-1297.27668	0.36837	1.368
NQO-BF3	-1005.81627	0.15989	1.342	-1621.35839	0.37084	1.333
$ZnCl_2(NQO)_2$	-4062.24346	0.29486	1.315	-4677.78696	0.50554	1.311/1.313

Table S3. Free energy of individual or Lewis-acid bonded N-oxides and their complexes with pyrene ion

	G (Ind),[a] a.u.	G (Comp),[b] a.u.	Δ G,[c] kcal/mol
NPO	-527.672204	-1143.013294	1.69
NPO-H^+	-528.072921	-1143.42181	-3.20
NPO-BF3	-852.16301	-1467.50681	-0.01
$ZnCl_2(NPO)_2$	-3754.937415	-4370.280444	0.47
NQO	-681.208796	-1296.553289	-0.45
NQO-H	-681.610838	-1296.959225	-2.89
NQO-BF3	-1005.698888	-1621.043225	-0.35
$ZnCl_2(NQO)_2$	-4062.011677	-4677.358153	-1.69

a) Free energy of individual or Lewis-acid bonded N-oxides; free energy of pyrene is 615.343782 a.u.

b) Free energy of the corresponding complexes with pyrene. c) Free energy change of complex formation.

Table S4. Crystallographic, data collection, and structure refinement details

**Crystals**	**1**<br>$ZnCl_2$-NPO	**2**<br>$(ZnCl_2(NPO)_2)_2$ $(pyrene)_5$	**3**<br>$(ZnCl_2(NQO)_2)_2$ $(pyrene)_3$	**4**<br>$ZnCl_2(THF)(NQO)_2)$· $(pyrene)_3$	**5**<br>I_9·2(NPO-H-NPO) ·pyrene
Chemical formula	$C_5H_4Cl_2N_2O_3Zn$	$2(C_{10}H_8Cl_2N_4O_6Zn)$ $\cdot 5(C_{16}H_{10})$	$2(C_{18}H_{12}Cl_2N_4O_6Zn)$ $\cdot 3(C_{16}H_{10})$	$C_{13}H_{14}Cl_2N_2O_4Zn\cdot$ $1.5(C_{16}H_{10})$	$I_9\cdot C_{16}H_{10}\cdot$ $2(C_5H_{4.5}N_2O_3)$
M_r	276.39	1844.14	1639.89	701.89	1625.55
Crystal system, space group	Monoclinic, $P2_1/c$	Triclinic, $P\bar{1}$	Triclinic, $P\bar{1}$	Monoclinic, $P2_1/c$	Monoclinic, $C2/c$
Temperature (K)	150	150	150	150	150
a, *b*, *c* (Å)	13.422 (2), 5.7857 (9), 11.8138 (18)	8.1143 (2), 10.5144 (3), 23.9214 (7)	10.0729 (7), 11.3897 (7), 17.1883 (9)	7.6416 (15), 10.372 (2), 38.676 (6)	12.9004 (7), 15.6853 (9), 19.0694 (11)
α, β, γ (°)	108.497 (3)	86.4101 (15), 87.8931 (16), 87.8871 (16)	100.530 (2), 97.352 (3), 112.962 (3)	90.27 (1)	97.055 (2)
V (Å^3)	870.0 (2)	2034.30 (10)	1741.35 (19)	3065.2 (10)	3829.4 (4)
Z	4	1	1	4	4
Radiation type	Cu *K*α	Mo *K*α	Mo *K*α	Mo *K*α	Mo *K*α
μ (mm^{-1})	9.42	0.80	0.92	1.02	7.33
Crystal size (mm)	0.15 × 0.12 × 0.02	0.45 × 0.32 × 0.30	0.29 × 0.22 × 0.13	0.42 × 0.38 × 0.26	0.55 × 0.35 × 0.05
Colour	Pale-yellow	red	red	dark red	black
Diffractometer	Bruker AXS D8 Quest				
Absorption correction	Multi-scan *SADABS* 2016/2: Krause, L., Herbst-Irmer, R., Sheldrick G.M. & Stalke D., J. Appl. Cryst. 48 (2015) 3-10				
T_{min}, T_{max}	0.501, 0.754	0.668, 0.747	0.671, 0.747	0.701, 0.747	0.448, 0.747
No. of measured, independent and observed [$I > 2\sigma(I)$] reflections	11431, 1870, 1832	74538, 15547, 12913	44957, 12976, 9339	92200, 11729, 10851	104467, 7342, 6142
R_{int}	0.037	0.035	0.063	0.026	0.054
$(\sin \theta/\lambda)_{max}$ (Å^{-1})	0.639	0.770	0.771	0.771	0.771
$R[F^2 > 2\sigma(F^2)]$, $wR(F^2)$, S	0.021, 0.058, 1.09	0.032, 0.088, 1.04	0.037, 0.093, 1.02	0.028, 0.076, 1.10	0.033, 0.086, 1.09
No. of reflections	1870	15547	12976	11729	7342
No. of parameters	119	568	496	452	207
No. of restraints				160	
H-atom treatment	H-atom parameters constrained				mixed [b)]
$\Delta\rho_{max}$, $\Delta\rho_{min}$ (e Å^{-3})	0.37, -0.32	0.50, -0.46	0.49, -0.43	0.54, -0.46	1.01, -1.13

a) A THF molecule was refined as disordered. The oxygen atom was omitted from the disorder. The two disordered moieties were restrained to have similar geometries. Uij components of ADPs for disordered atoms closer to each other than 2.0 Angstrom were restrained to be similar. Subject to these conditions the occupancy ratio refined to 0.497(8) to 0.503(8). b) H atoms treated by a mixture of independent and constrained refinement $w = 1/[\sigma^2(F_o^2) + (0.0251P)^2 + 43.2911P]$ where $P = (F_o^2 + 2F_c^2)/3$

Atomic coordinates

NPO

O	-3.35597100	0.00004000	0.00010200
O	2.68255700	1.07972300	-0.00052100
O	2.68259900	-1.07968100	0.00048800
N	-2.09169000	0.00003500	-0.00003900
N	2.11718100	0.00000500	0.00009300
C	-1.40256400	1.18209900	0.00015200
H	-2.03296100	2.06001400	0.00031600
C	-0.02661600	1.20648800	0.00017900
H	0.50127700	2.15147700	0.00026400
C	0.66034500	-0.00001100	0.00002400
C	-0.02667300	-1.20657800	-0.00019000
H	0.50125300	-2.15154900	-0.00034400
C	-1.40255700	-1.18215100	-0.00027200
H	-2.03310000	-2.05996200	-0.00052400

$NPO\text{-}H^+$

H	-3.68760600	-0.00013200	-0.81035300
O	-3.33835100	-0.00005400	0.10375200
O	2.72045100	-1.08236100	0.00053500
O	2.72047100	1.08234300	-0.00069500
N	-1.97584500	0.00007500	-0.00018000
N	2.17565800	-0.00000900	-0.00220600
C	-1.35090300	-1.18931100	0.00412100
H	-1.99016900	-2.06240800	0.02050700
C	0.03077800	-1.21663300	-0.00466600
H	0.56698800	-2.15665100	-0.00152300
C	0.69278000	0.00004400	-0.00672100
C	0.03083700	1.21660500	-0.00412700
H	0.56665900	2.15683200	-0.00011100
C	-1.35100600	1.18927600	0.00444700
H	-1.99004400	2.06258800	0.02112900

$BF_3\cdot NPO$

O	-1.96498700	-0.00040700	-1.01434900
O	3.93557700	-1.08074800	0.33892300
O	3.93484900	1.08105200	0.34123700
N	-0.66238400	-0.00027200	-0.69832500
N	3.39667900	0.00009400	0.22130400
C	-0.04477600	-1.18439900	-0.54518200
H	-0.67204700	-2.05487300	-0.67722500
C	1.30345400	-1.21424200	-0.24193700
H	1.82450900	-2.15471900	-0.12106900
C	1.95326600	-0.00002700	-0.09762200
C	1.30350000	1.21410800	-0.24309900
H	1.82457900	2.15467800	-0.12310700
C	-0.04468000	1.18401900	-0.54636800
H	-0.67201000	2.05433200	-0.67933100
B	-2.81645300	0.00009200	0.28307000
F	-2.46985600	1.14176900	0.98417100
F	-4.10828300	-0.00016900	-0.16441400
F	-2.46974200	-1.14099600	0.98505300

$ZnCl_2(NPO)_2$

Zn	-0.10417600	1.72722700	0.16367600
Cl	-1.22419600	3.57442200	-0.51360300
Cl	-0.33629700	0.70299800	2.18425100
O	-0.69421400	0.25848700	-1.10187100
O	-6.51879400	-1.38964200	-0.03456600
O	-5.48507400	-3.12687300	0.72887200
N	-1.84817000	-0.28190800	-0.77595100
N	-5.51481800	-2.02507600	0.21791600
C	-2.98693200	0.40017700	-1.03017100
H	-2.84461900	1.37754600	-1.47476900
C	-4.21156700	-0.15070800	-0.70796100
H	-5.12754400	0.39040000	-0.90565900
C	-4.22326000	-1.40842100	-0.12761800
C	-3.05458100	-2.10214700	0.13813200
H	-3.07010700	-3.08110700	0.59864400
C	-1.85714300	-1.50089100	-0.19716200
H	-0.88111700	-1.93497200	-0.02968100
O	1.81553400	1.91808100	-0.31361800
O	6.51897400	-1.94413400	0.51770500
O	5.24013100	-3.17256200	-0.71727300
N	2.66506400	0.92206800	-0.23988600
N	5.50129900	-2.15162500	-0.11232700
C	3.80784900	1.11033600	0.45620100
H	3.89666100	2.07746000	0.93139500
C	4.76026700	0.11240000	0.51784800
H	5.67814600	0.26133300	1.07138900
C	4.50034800	-1.07377900	-0.14828000
C	3.32383200	-1.27024900	-0.85140200
H	3.12325500	-2.19895600	-1.36938000
C	2.40620600	-0.23891300	-0.88280800
H	1.45279800	-0.28253300	-1.39617300

NQO

O	-2.40862800	-2.19399500	0.07541400
O	2.66582800	1.38891100	0.45041800
O	3.43427600	-0.44775400	-0.36983100
N	-1.29061000	-1.60638300	0.03984700
N	2.51815300	0.25666400	0.02031900
C	-0.14757800	-2.31097900	0.02433400
H	-0.27884100	-3.38338000	0.04478000
C	1.09275800	-1.67888300	-0.00382000
H	1.99189600	-2.28110700	-0.01072500
C	1.17406900	-0.31090200	-0.00375300
C	-0.00607600	0.49159300	-0.00886800
C	-0.06224400	1.90961300	-0.07465100
H	0.85713900	2.47417400	-0.10783500
C	-1.27116300	2.56184600	-0.10134100
H	-1.28590200	3.64523100	-0.15588700
C	-2.48823300	1.84972000	-0.06078600
H	-3.43126000	2.38485000	-0.07823500
C	-2.47796500	0.47859800	-0.00942500
H	-3.38512100	-0.11011400	0.01076000
C	-1.24565400	-0.20042400	0.00963800

NQO-H^+

N	1.30735900	-1.47224100	0.04409100
N	-2.54459300	0.13202600	0.02076000
O	2.54282700	-2.04921600	0.14767700
O	-2.76165800	1.13526900	0.66793800
O	-3.36395400	-0.51525900	-0.59450700
C	1.24099100	-0.09690000	0.00864500
C	2.41864300	0.67213200	-0.00346000
H	3.38303600	0.18497000	0.04526900
C	2.30179800	2.03815500	-0.07235800
H	3.19765500	2.64859200	-0.08446600
C	1.03310900	2.66049500	-0.13222700
H	0.97300100	3.74055900	-0.20005600
C	-0.12083100	1.92152300	-0.10011400
H	-1.08513100	2.40802500	-0.13642200
C	-0.05165000	0.50510900	-0.01911100
C	-1.15579300	-0.38049000	0.00781200
C	-1.02628600	-1.74439200	0.03598400
H	-1.88884100	-2.39751000	0.04219800
C	0.26071300	-2.28227300	0.06874400
H	0.47793300	-3.34203500	0.11519700
H	2.86109400	-2.18760100	-0.76801800

NQO-BF3

N	0.83887100	-0.40728400	-0.64005200
N	-3.20721600	-1.05073100	0.22433100
O	2.14003400	-0.24678300	-0.92762500
O	-3.71974200	-1.98249100	-0.36093800
O	-3.74667700	-0.36128700	1.06689900
C	0.34839800	-1.63531300	-0.63320300
H	1.05816400	-2.42429800	-0.83865900
C	-1.00855500	-1.84645800	-0.36625600
H	-1.41181400	-2.85042400	-0.36855100
C	-1.79488700	-0.76166800	-0.09404900
C	-1.30154800	0.56774000	-0.10656200
C	-2.06876200	1.74467100	0.10384200
H	-3.12645700	1.66252000	0.30947700
C	-1.46654700	2.97425000	0.03930800
H	-2.06138000	3.86713000	0.19582200
C	-0.08268200	3.09823800	-0.22735400
H	0.36886200	4.08327800	-0.26544100
C	0.69403100	1.98977200	-0.44510200
H	1.75206700	2.05646300	-0.65475400
C	0.08465200	0.72044000	-0.39126600
B	3.01444300	-0.46980500	0.33296400
F	2.60377400	0.45043900	1.27918300
F	4.29221500	-0.26555500	-0.11400000
F	2.78325900	-1.76544900	0.76420400

$ZnCl_2(NQO)_2$

Zn	0.01059500	-1.60763600	-0.16591700
Cl	0.83509800	-0.40545000	-1.92304600
Cl	-1.29099100	-3.44721500	-0.43502100
O	-1.06405700	-0.49675600	1.13114200
O	-6.68136300	0.89653800	-1.14229500
O	-7.17099600	-0.14783300	0.68167200

O	1.54891000	-1.69538500	1.11444200
O	7.20187300	-0.14793200	-0.69142300
O	6.23415700	1.73382000	-1.11487500
N	-2.32386100	-0.26949200	0.83071300
N	-6.38129200	0.33605000	-0.10534300
N	2.66250100	-1.12442600	0.71156200
N	6.24004500	0.59506700	-0.68775100
C	-3.24542800	-1.14961200	1.19571300
H	-2.87416200	-2.02523300	1.71014300
C	-4.59655500	-0.92948500	0.90135700
H	-5.33763900	-1.65582200	1.20782700
C	-4.95064500	0.19560700	0.21292000
C	-3.99564300	1.17040500	-0.18613200
C	-4.27503300	2.39434400	-0.84942100
H	-5.29494900	2.63815300	-1.10903100
C	-3.25609300	3.26053300	-1.15706700
H	-3.48552600	4.19236200	-1.66210700
C	-1.91569500	2.95800300	-0.82935800
H	-1.12657000	3.65414800	-1.09110800
C	-1.60312900	1.79290700	-0.17586400
H	-0.58596700	1.53746700	0.09083400
C	-2.64166800	0.89921700	0.15197100
C	3.58536400	-1.86122800	0.11131200
H	3.33560400	-2.90643100	-0.01263700
C	4.78412400	-1.28951500	-0.32794500
H	5.52893000	-1.90755100	-0.81150200
C	4.97963500	0.05144800	-0.15468000
C	4.01991200	0.87687400	0.49034700
C	4.15629100	2.26509800	0.75967800
H	5.05398000	2.78094800	0.45176300
C	3.15569000	2.94449900	1.40646200
H	3.27506600	4.00377800	1.60545900
C	1.97472100	2.28640900	1.81818400
H	1.19526000	2.84587500	2.32433300
C	1.80925700	0.94270000	1.59579000
H	0.91801000	0.41421800	1.91163100
C	2.83162100	0.23387400	0.93264700

NPO·pyrene

O	-3.67902900	1.17481300	-1.03215400
O	2.42205700	1.71422900	-1.34079200
O	2.06568800	2.60374600	0.59421900
N	-2.43945600	1.39562500	-0.89806000
N	1.68769000	2.07466000	-0.43796400
C	-1.56166600	1.00143800	-1.87143000
H	-2.02798100	0.50228100	-2.70936800
C	-0.21039400	1.22556700	-1.75007700
H	0.47041100	0.89143200	-2.52263300
C	0.25711600	1.85783800	-0.60449200
C	-0.62096600	2.26907300	0.38742500
H	-0.26272900	2.75736200	1.28444400
C	-1.96699800	2.02704200	0.21723900
H	-2.73381800	2.29651900	0.92961400
C	-0.75267500	-2.32881100	-1.58649800
H	-1.43101200	-2.76462300	-2.31536100
C	0.57843400	-2.26881400	-1.83289300

H	0.98677600	-2.65875500	-2.76153200
C	1.49174500	-1.68778200	-0.87938800
C	2.86711300	-1.60362600	-1.11971900
H	3.26642300	-1.99639000	-2.05086000
C	3.71734800	-1.01883200	-0.18431700
H	4.78123800	-0.95594400	-0.38945000
C	3.21195200	-0.50151200	1.00316100
H	3.87613500	-0.03008000	1.72227700
C	1.84140200	-0.56986500	1.28500300
C	0.96920900	-1.17425200	0.33958100
C	1.27920500	-0.02141100	2.49273100
H	1.95193100	0.45548200	3.20042600
C	-0.05137000	-0.09340700	2.74550200
H	-0.46335400	0.32451400	3.66038500
C	-0.95782100	-0.70777700	1.80950600
C	-2.34105400	-0.74955200	2.02973500
H	-2.74282800	-0.33334700	2.94981400
C	-3.19401800	-1.29955200	1.07873400
H	-4.26466600	-1.30853800	1.25551400
C	-2.68664600	-1.82935900	-0.10484700
H	-3.35951700	-2.24825900	-0.84800200
C	-1.31151500	-1.81280000	-0.36074300
C	-0.43413000	-1.24126400	0.60190300

H-NPO^{+}·pyrene

O	-3.40953900	1.49322800	-1.01480000
H	-3.65085900	0.62933400	-0.60721800
O	2.73944600	1.46952100	-1.31590000
O	2.46025400	2.35364100	0.64002800
N	-2.05502300	1.57985300	-0.85603300
N	2.05801600	1.88436200	-0.40385300
C	-1.28073800	1.14735100	-1.86843100
H	-1.80289000	0.73067700	-2.72081200
C	0.09054700	1.25547200	-1.75186600
H	0.74556200	0.90588200	-2.53953200
C	0.58911000	1.80068900	-0.57749100
C	-0.22317600	2.26997600	0.44081700
H	0.18711300	2.70728500	1.34149100
C	-1.59069700	2.14440500	0.26964800
H	-2.33779200	2.46774100	0.98305400
C	-0.94254100	-2.18140900	-1.68823800
H	-1.61724500	-2.54192800	-2.45953500
C	0.40087200	-2.22519600	-1.86968200
H	0.81903500	-2.62070900	-2.79106900
C	1.31065800	-1.75555000	-0.85569000
C	2.69867100	-1.77730200	-1.03149500
H	3.10927900	-2.17038000	-1.95703700
C	3.54741000	-1.29491200	-0.03836200
H	4.62101300	-1.31324400	-0.19304200
C	3.03015200	-0.77907600	1.14586100
H	3.69626900	-0.39251100	1.91169800
C	1.64692400	-0.73857300	1.36153300
C	0.77469900	-1.23548900	0.35499800
C	1.07501400	-0.18167700	2.56129300
H	1.75035600	0.20627500	3.31862900
C	-0.26746600	-0.14055200	2.74990800

H	-0.68596600	0.27840400	3.66057100
C	-1.17633600	-0.64543700	1.75085700
C	-2.56645200	-0.58286100	1.91202600
H	-2.97804500	-0.17094800	2.82889900
C	-3.41849200	-1.05926300	0.91474100
H	-4.49373400	-1.03176100	1.06772600
C	-2.90053700	-1.58796600	-0.26884100
H	-3.56932600	-1.96775600	-1.03698400
C	-1.51278700	-1.66086300	-0.47053100
C	-0.64067000	-1.18793200	0.54856800

BF_3-NPO·pyrene

O	-2.82556000	0.41445100	0.01106300
O	2.42471500	-2.06885800	2.02996900
O	1.89509100	-3.49617400	0.49610600
N	-1.74785400	-0.31994600	0.32197200
N	1.69562200	-2.49911300	1.15956700
B	-4.04859500	-0.07508700	0.82451700
F	-5.06922400	0.74186800	0.41986900
F	-3.72325700	0.06615400	2.16274100
F	-4.24089000	-1.40626400	0.49698200
C	-0.96734500	0.09254800	1.33472600
H	-1.30105100	0.98576800	1.84394700
C	0.18387600	-0.61355100	1.63727800
H	0.84682600	-0.28189800	2.42707700
C	0.46212500	-1.73859100	0.87909100
C	-0.36617400	-2.16752400	-0.14597800
H	-0.13429900	-3.04799100	-0.73068100
C	-1.49787400	-1.41957800	-0.41019800
H	-2.22108700	-1.63639200	-1.18361700
C	1.40238000	-0.36174500	-2.10187500
C	0.60381700	-0.84479100	-3.14586400
H	0.92218400	-1.72980700	-3.69003000
C	-0.59179000	-0.21098900	-3.47552000
H	-1.20426000	-0.60286200	-4.28134100
C	-1.01258500	0.91638800	-2.77457100
H	-1.95301300	1.39868400	-3.02552100
C	-0.23610100	1.43861400	-1.73245100
C	-0.65877200	2.58308100	-0.96513600
H	-1.59706600	3.06070900	-1.23165900
C	0.08348700	3.04568700	0.07146300
H	-0.24846700	3.90558400	0.64690200
C	1.31542900	2.40332400	0.45608900
C	2.05965100	2.82383300	1.56459500
H	1.71963000	3.68390400	2.13477500
C	3.21402900	2.14379000	1.94412400
H	3.77498700	2.47820600	2.81082700
C	3.64979600	1.03428200	1.22574300
H	4.54349000	0.49847100	1.53307300
C	2.93865900	0.58656500	0.10599100
C	3.33992200	-0.58063800	-0.63820800
H	4.23721900	-1.10694400	-0.32294600
C	2.61057200	-1.03130200	-1.68931500
H	2.91676900	-1.92133000	-2.23236500
C	0.98308000	0.79390200	-1.38658300
C	1.75933500	1.27562300	-0.28760600

$ZnCl_2(NPO)_2$·pyrene

Zn	1.94718700	-2.02220800	0.59837000
Cl	3.16280500	-3.51052600	-0.59361300
Cl	2.44120000	-1.07775700	2.62987800
O	1.96330500	-0.28526300	-0.47383700
O	7.57237100	2.20633200	-0.83060600
O	6.62387800	3.59464500	0.52673200
N	3.08396800	0.39768400	-0.39537900
N	6.62799000	2.60602400	-0.17945600
C	4.16113300	-0.02171800	-1.09650500
H	4.01129000	-0.93193300	-1.66370900
C	5.34445000	0.68781300	-1.04154800
H	6.21100200	0.35683700	-1.59869800
C	5.38120900	1.82697400	-0.25409500
C	4.27806400	2.25318200	0.46597500
H	4.31435200	3.14060200	1.08395100
C	3.12145900	1.50327500	0.37868900
H	2.20166400	1.73287700	0.89885600
O	0.02765200	-2.47599800	0.45346400
O	-5.30588800	0.06144300	2.14363900
O	-3.96780900	1.71391300	2.52845800
N	-0.96038900	-1.72745800	0.87549500
N	-4.20486200	0.57752300	2.17020500
C	-2.18749100	-2.28948600	0.93002700
H	-2.22621700	-3.32338400	0.61628800
C	-3.27745900	-1.55547900	1.35798000
H	-4.26309000	-2.00066500	1.38998700
C	-3.06527300	-0.23896500	1.72970700
C	-1.80641700	0.33656800	1.67841800
H	-1.64681200	1.37133800	1.95241300
C	-0.75747300	-0.43652700	1.22619000
H	0.25458900	-0.07247900	1.10883800
C	-0.54967400	1.45082800	-1.48515600
H	0.52595900	1.48593400	-1.63163300
C	-1.22368900	2.52000700	-0.99479800
H	-0.69868500	3.43959700	-0.74932100
C	-2.64994600	2.47489900	-0.78227100
C	-3.36132800	3.55949100	-0.25785200
H	-2.82753000	4.47347900	-0.01224600
C	-4.73527000	3.47298800	-0.04634000
H	-5.27046000	4.32215400	0.36623000
C	-5.42422600	2.30260900	-0.34562100
H	-6.49297300	2.23334000	-0.16290200
C	-4.75015400	1.19346300	-0.87291200
C	-3.34945500	1.27783300	-1.10113800
C	-5.42557200	-0.04553100	-1.16463200
H	-6.49302300	-0.10474400	-0.97067600
C	-4.75394500	-1.11237500	-1.66464200
H	-5.27495400	-2.04132700	-1.88083400
C	-3.33554000	-1.05376000	-1.91209400
C	-2.61498200	-2.15918400	-2.38384800
H	-3.14497800	-3.08307800	-2.59965600
C	-1.23713500	-2.08366000	-2.56210800
H	-0.69015000	-2.95303200	-2.91294100
C	-0.54882000	-0.90559700	-2.28227200
H	0.52962900	-0.85669900	-2.40090800

C	-1.23221300	0.22202900	-1.81242500
C	-2.63997700	0.15053100	-1.61997800

NQO·pyrene

O	1.47758900	-2.59082700	1.84542500
O	-1.50857600	2.85196200	1.15603100
O	0.47429400	3.42751400	1.74790200
N	1.01187400	-1.41614400	1.82299000
N	-0.37453200	2.58024100	1.51642000
C	1.82565600	-0.35852200	1.97046600
H	2.86762000	-0.60641900	2.11172800
C	1.34000800	0.94103200	1.88738300
H	2.03401800	1.76621900	1.97500900
C	0.00742500	1.18017800	1.65767600
C	-0.91519800	0.09319300	1.54811900
C	-2.32282600	0.18212100	1.37213200
H	-2.78500900	1.15242800	1.29048600
C	-3.09458600	-0.95248600	1.30181900
H	-4.16654300	-0.85108200	1.16526800
C	-2.52391700	-2.23814000	1.39574900
H	-3.15324100	-3.11964100	1.33622400
C	-1.16920100	-2.36990100	1.56199300
H	-0.68145900	-3.33262600	1.63488600
C	-0.36672800	-1.21577000	1.64013800
C	1.69477400	-1.37189800	-1.22855600
C	3.06262600	-1.45354300	-0.93793300
H	3.51819800	-2.43166100	-0.81150400
C	3.82785500	-0.29937400	-0.80371700
H	4.88708400	-0.37863600	-0.57934700
C	3.24542500	0.95821300	-0.94890000
H	3.84729500	1.85613400	-0.83636200
C	1.88061300	1.08348600	-1.23113400
C	1.23495900	2.36787000	-1.34360400
H	1.84121800	3.26116200	-1.21890400
C	-0.09604900	2.46617100	-1.58166300
H	-0.57519500	3.43951900	-1.64593700
C	-0.91770500	1.29176100	-1.73807600
C	-2.29470700	1.37232100	-1.97572900
H	-2.76259900	2.35090400	-2.04239300
C	-3.05670100	0.21768700	-2.12811900
H	-4.12293700	0.29717700	-2.31617200
C	-2.46481400	-1.03789900	-2.02924000
H	-3.06674100	-1.93682600	-2.13479500
C	-1.09370500	-1.16310400	-1.77634200
C	-0.45384300	-2.44563700	-1.63057000
H	-1.06815900	-3.33758200	-1.72336300
C	0.87081500	-2.54612900	-1.36041500
H	1.33939600	-3.51760700	-1.23149000
C	1.09298600	-0.09253400	-1.37332100
C	-0.30662600	0.01268400	-1.63883200

H-NQO^{+}·pyrene

N	1.46816900	-0.30980600	1.90417000
N	-1.00203700	2.89730700	0.83119700
O	2.33810400	-1.33347100	2.16323500
O	-2.14456800	2.71934700	0.46335800

O	-0.44705400	3.97520600	0.88399300
C	0.12248500	-0.59865700	1.86637700
C	-0.31976000	-1.89945100	2.17040000
H	0.40117100	-2.67199400	2.40000100
C	-1.66743000	-2.15508300	2.14270600
H	-2.02628900	-3.15365500	2.36664200
C	-2.58857100	-1.13469900	1.82173100
H	-3.64913100	-1.35986300	1.80735500
C	-2.17094400	0.13688600	1.51955600
H	-2.89236900	0.89863200	1.27239100
C	-0.78560200	0.45365900	1.52415300
C	-0.19552400	1.71435500	1.23225600
C	1.15716700	1.93331900	1.30003100
H	1.58023500	2.90293800	1.07578200
C	1.99718400	0.87309600	1.63626100
H	3.07574600	0.94218400	1.69316400
C	-0.92009900	-1.96610300	-1.25378600
C	0.02942100	-2.98612700	-0.89039800
H	-0.34422600	-3.98723200	-0.69185100
C	1.35253200	-2.70967500	-0.77546900
H	2.05768500	-3.48416400	-0.48712000
C	1.86247200	-1.38791800	-1.03530400
C	3.22388100	-1.07707200	-0.90850000
H	3.91845200	-1.85703400	-0.60972100
C	3.68245200	0.21227700	-1.15703000
H	4.73942800	0.43723500	-1.05606300
C	2.79552400	1.21968500	-1.53739900
H	3.16046100	2.22489900	-1.73020900
C	1.42807300	0.95159000	-1.67387900
C	0.47716500	1.97684500	-2.02041900
H	0.84760500	2.98059300	-2.21182100
C	-0.85090800	1.70562000	-2.09672900
H	-1.55966700	2.49090700	-2.34528100
C	-1.36204600	0.38303900	-1.84418500
C	-2.73023100	0.08863400	-1.90372300
H	-3.43101400	0.88193400	-2.14867400
C	-3.18772100	-1.20249400	-1.65954700
H	-4.25019400	-1.41658400	-1.71515000
C	-2.29442600	-2.22124100	-1.33826200
H	-2.65858300	-3.22671100	-1.14441500
C	-0.44482100	-0.65134400	-1.51238200
C	0.95107600	-0.36389900	-1.41392900
H	2.42780500	-1.37469700	3.13753900

BF_3-NQO·pyrene

N	-1.58510800	-0.35022200	-0.77645800
N	1.83673200	1.55678700	-2.31549000
O	-2.63194800	-0.94615700	-0.20614300
O	2.92598300	1.01804700	-2.29708300
O	1.63838400	2.69249300	-2.70100600
B	-4.03563400	-0.37843400	-0.53402800
F	-4.06212600	-0.10829200	-1.88681200
F	-4.21767500	0.78154200	0.20866500
F	-4.87730400	-1.38505200	-0.14749800
C	-0.50481300	-1.17009800	-1.05220300
C	-0.62389000	-2.55255700	-0.80169400

H	-1.55062600	-2.93412700	-0.39742800
C	0.44259300	-3.36876600	-1.07644000
H	0.36516900	-4.43420900	-0.88945100
C	1.63670600	-2.83198000	-1.60332400
H	2.47186100	-3.49135700	-1.81401300
C	1.76606600	-1.48947400	-1.85512200
H	2.68764900	-1.10168300	-2.25767000
C	0.68999000	-0.60160000	-1.58533300
C	0.66626600	0.80486700	-1.81239900
C	-0.44779900	1.55633600	-1.56202600
H	-0.45183200	2.62295700	-1.73989400
C	-1.58359700	0.95145800	-1.01465800
H	-2.47210000	1.49843700	-0.73175900
C	1.52577700	-1.44029800	1.80326700
C	0.28265100	-1.97000800	2.30303100
H	0.23216300	-3.03197800	2.52889000
C	-0.80686300	-1.17911900	2.46333600
H	-1.74665700	-1.59326600	2.81740800
C	-0.75725400	0.22845500	2.16014600
C	-1.88249900	1.05341800	2.28275500
H	-2.82389400	0.62244700	2.60928300
C	-1.80643500	2.40512200	1.96190200
H	-2.69034700	3.02845700	2.05292800
C	-0.61029500	2.96161700	1.51179200
H	-0.55928300	4.01743300	1.25919200
C	0.53343100	2.16808900	1.36154900
C	1.76888400	2.69402100	0.83681900
H	1.81340800	3.75054500	0.58581000
C	2.84671400	1.89362200	0.64330700
H	3.76692900	2.29749900	0.22941000
C	2.80354600	0.48815900	0.96099000
C	3.89873600	-0.35781600	0.74865000
H	4.81273500	0.05653400	0.33192900
C	3.82229500	-1.70998300	1.06906900
H	4.68178700	-2.35184000	0.90296600
C	2.64955000	-2.24794700	1.59129600
H	2.59157900	-3.30692600	1.82918400
C	1.60256700	-0.05476100	1.49289600
C	0.46148000	0.78398500	1.68266800

$ZnCl_2(NQO)_2$·pyrene

Zn	1.76167800	-2.05002500	-0.49802200
Cl	2.94133300	-1.19431300	-2.24859700
Cl	1.10280400	-4.20924000	-0.30296400
O	0.25025000	-0.84983400	0.00738100
O	-5.61416500	-0.25428000	-2.06266700
O	-5.49351400	-2.32646400	-1.50436900
O	2.82130000	-1.43980800	1.09895700
O	8.48437100	0.79987500	0.35216700
O	7.43199700	2.23142900	-0.87234100
N	-0.96570700	-0.95228300	-0.47065600
N	-4.99971500	-1.22638000	-1.66601000
N	3.91473900	-0.76260600	0.83385400
N	7.47039400	1.28053900	-0.11491200
C	-1.68826800	-2.03141200	-0.20575300
H	-1.18876300	-2.80298300	0.36579400

C	-3.01497700	-2.11393600	-0.63914600
H	-3.59866200	-2.99025400	-0.39198100
C	-3.57227900	-1.07128400	-1.32706600
C	-2.81440500	0.08840900	-1.66904000
C	-3.25593700	1.20734500	-2.42515200
H	-4.27055400	1.23740000	-2.78780100
C	-2.40013100	2.24743800	-2.69176900
H	-2.76141300	3.09159500	-3.26957700
C	-1.06559400	2.23899100	-2.23337300
H	-0.40730100	3.06934100	-2.46537000
C	-0.59543000	1.18122200	-1.49882100
H	0.42364200	1.13470300	-1.13734200
C	-1.46444600	0.10913400	-1.21272100
C	5.07173700	-1.40395500	0.75907700
H	5.02095300	-2.47225500	0.92143300
C	6.25460900	-0.71456500	0.47144800
H	7.19043100	-1.25464100	0.41414700
C	6.19816000	0.63120400	0.24186100
C	4.98083000	1.35944400	0.32332400
C	4.82955100	2.76192100	0.15126700
H	5.69706300	3.36685200	-0.06892900
C	3.59162600	3.34206800	0.26195800
H	3.49256200	4.41348600	0.12713800
C	2.44550900	2.56497000	0.54577000
H	1.47244100	3.04182900	0.61339900
C	2.54894400	1.21143200	0.74139600
H	1.68547500	0.59436900	0.96150900
C	3.81729800	0.60593800	0.63525000
C	-1.71935800	0.38407200	2.28067500
C	-1.18089100	-0.74091000	2.91913600
H	-0.13757400	-0.72831000	3.22288600
C	-1.96600400	-1.86547900	3.15241700
H	-1.53395100	-2.73024200	3.64601000
C	-3.30208900	-1.89270500	2.75462400
H	-3.90945300	-2.77466700	2.93973100
C	-3.87469900	-0.79455100	2.10319700
C	-5.23806300	-0.80562900	1.63366300
H	-5.83960700	-1.69116200	1.82128300
C	-5.75759500	0.25080000	0.96059000
H	-6.78088400	0.22352400	0.59544400
C	-4.97109800	1.42939600	0.69375600
C	-5.48337900	2.52213400	-0.01573600
H	-6.50549400	2.48116500	-0.38229700
C	-4.69946500	3.64877900	-0.24640500
H	-5.11327400	4.48967800	-0.79398000
C	-3.38738300	3.70323100	0.21434700
H	-2.77748400	4.58324700	0.02741900
C	-2.83102800	2.62483300	0.91237100
C	-1.47091400	2.63756500	1.38667300
H	-0.87972700	3.53326900	1.21206900
C	-0.93825700	1.56871900	2.02967700
H	0.08779500	1.59170300	2.38856300
C	-3.07505900	0.35577800	1.85423600
C	-3.62931100	1.47481600	1.15971600

Pyrene

C	-1.42542000	1.23083300	0.00000200
C	-2.82509000	1.20858700	-0.00000100
H	-3.36937100	2.14906700	0.00000500
C	-3.51531400	-0.00000700	-0.00001000
H	-4.60077300	-0.00000400	-0.00002100
C	-2.82509500	-1.20858700	-0.00000900
H	-3.36936700	-2.14907300	-0.00000700
C	-1.42540900	-1.23083600	-0.00000400
C	-0.67748200	-2.46326600	-0.00000900
H	-1.23029100	-3.39884600	-0.00002000
C	0.67748600	-2.46326300	-0.00000400
H	1.23029600	-3.39884300	-0.00001400
C	1.42540800	-1.23083100	0.00000400
C	2.82510000	-1.20858500	0.00001500
H	3.36935800	-2.14907900	0.00003500
C	3.51531400	-0.00001100	0.00000900
H	4.60077300	0.00001000	0.00001600
C	2.82508500	1.20858800	-0.00000500
H	3.36937900	2.14906100	-0.00002400
C	1.42542200	1.23083800	-0.00000300
C	0.67747700	2.46327400	0.00000500
H	1.23029800	3.39884700	0.00001200
C	-0.67748000	2.46327200	0.00001000
H	-1.23030400	3.39884400	0.00002200
C	-0.71419900	-0.00000100	0.00000000
C	0.71419900	-0.00000200	0.00000100
